# A Hadean timeline for the emergence of the RNA World

**DOI:** 10.1038/s41467-026-76978-3

**Published:** 2026-09-22

**Authors:** Oleg Abramov, Anna Medvegy, Barbara Kremer, Stephen J. Mojzsis

**Affiliations:** 1 Bayerisches Geoinstitut (BGI), University of Bayreuth, Universitätsstrasse 30, 95447, Bayreuth, Germany; 2 Planetary Science Institute, 1700 East Fort Lowell Road, Suite 106, Tucson, Arizona, 85719 USA; 3 HUN-REN Research Centre for Astronomy and Earth Sciences (CSFK), MTA Centre of Excellence, Konkoly-Thege Miklós út 15-17, 1121, Budapest, Hungary; 4 Department of Plant Systematics, Ecology and Theoretical Biology, Institute of Biology, Eötvös Loránd University (ELTE), Pázmány Péter sétány 1/C, 1053, Budapest, Hungary; 5 Institute of Paleobiology, Polish Academy of Sciences, Twarda 51/55, 00-818, Warsaw, Poland; 6 Department of Earth Science, University of Colorado, 2200 Colorado Avenue, UCB 399, Boulder, Colorado, 80309 USA

**Keywords:** Astrobiology, Origin of life

## Abstract

Estimates for when life emerged on Earth range from ca. 3.8 Ga (Eoarchean) to the Hadean eon (≥4.0-4.5 Ga). Here, we conclude that a proposed early stage of life – the RNA World – appeared at ca. 4.33 Ga by tracking the near-surface thermal stability of key biomolecules comprising postulated RNA organisms. We apply a global three-dimensional numerical model to analyse the thermal evolution profile of Hadean crust in response to late accretion bombardment. Results show that frequent global sterilization events persisted up until about 4.4 Ga owing to hot ejecta deposition, rock vapour condensation, and enhanced geothermal gradients. Later, conditions became favorable to persistent biochemistry by supporting interconnected zones of biomolecule stability associated with extensive energy-rich hydrothermal oases. We identify an optimal time for the RNA World that dovetails with the emergence of the Last Universal Common Ancestor (LUCA) population.

## Abstract (web summary)

A 3-D thermal model identifies a ~ 4.33 Ga temporal “sweet spot” for life’s origin, when decreased bombardment enabled mild hydrothermal niches to support RNA World chemistry and stable biomolecule preservation, congruent with LUCA estimates.

## Introduction

The origin of life has roots in the earliest history of our planet. The timing of its emergence, however, is the subject of active debate and conjecture across disciplines spanning prebiotic chemistry, molecular biology, astrophysics and planetary geodynamics, early Earth geochemistry, evolutionary theory and bioinformatics. Disparate as they are, these various approaches to the problem of both where and when abiogenesis occurred share a common premise: For life to arise, the appropriate prebiotic building blocks must be present in sufficient quantity and concentration to react. At the same time, these reactants must be kept in an environment favoring their own production, stability and persistence so that they may ultimately lead to functional biochemistry^1^. Thus, the global procreative reaction space which gave rise to life had to remain *biocompatible* long enough for it to become *habitable*. The latter term is used to imply that some fully functional biological entity with known properties has the potential survive in a particular environment, ‘extreme’ or otherwise. The former, is defined as a condition where biomolecules remain both available and stable for prebiotic chemistry to proceed, whether life actually arises or not.

At an unspecified time before DNA played a central role in biology, a precursor RNA World is thought to have existed^2^. The earliest lifeforms contained chiral RNA information-storage molecules which catalyzed their own replication without the need for other RNAs or encoded peptides^3–5^. Here, we focus on the thermal stability and persistence of the key building blocks (nucleotides, peptides, lipids) that made up an RNA organism on Hadean (pre-4 Ga) Earth. We show how tracking thermal stability for precursors to the RNA World serves frames our understanding of not only the durability of the first environments for productive and persistent prebiotic chemistry, but also the timing at which these environments became established.

### Rocks from above, fire from below

Judging from what we know of early solar system history and the conditions for planet formation, however, the Earth was initially globally inhospitable for maintaining the appropriate physical and chemical conditions leading to life^6,7^. This perceived limitation to biocompatibility of the early Hadean Earth is due in no small part to abundant evidence for a protracted history of intense post-primary accretion (i.e. late accretion) impacts by comets, leftover planetesimals and asteroids that affected the inner solar system^8,9^. As recorded in the cratered surfaces of the oldest solid planetary bodies and extrapolated to Earth, early bombardment sustained extreme surface conditions incompatible with stable liquid water, a prerequisite for life to begin. Consequently, abiogenesis was thwarted for an uncertain period of time. Despite many lingering uncertainties, it is now possible to place some bounds on the age of the biosphere from above, and from below.

From above, a hard upper limit to when life could start is without a doubt set by the time of the Moon-forming giant impact at ca. 4.51 Ga^10,11^. This event generated a deep global magma ocean, followed by a protracted history of cooling and remelting involving intermittent hot, dense atmospheres modulated by vapourised rock and volatiles^12^. After the Moon, other large (>500 km diameter) impacts continued to sporadically vapourise the first oceans and produce transient steam atmospheres raising surface temperatures above 300 °C for millennia; such conditions sterilized any latent biosphere or otherwise hampered prebiotic chemistry^13,14^. Not least, ejecta blankets and ballistic fallout from regional- or hemispheric-scale impacts buried the surface under layers of hot and molten rock hundreds of meters thick to destroy any potential for even deep subsurface biocompatible niches^15,16^. Dynamical models coupled to cratering records indicate that ocean-sterilizing impacts are expected to have lasted until ~4.4 Ga^8,9,17^.

From below, high mantle temperatures (>1700 °C) in Hadean Earth’s interior drove vigorous convection, which in turn recycled primordial crust and exposed the shallow subsurface to extreme heat flux^18^. Whilst these conditions appear to have delayed continuous availability and stability of biomolecules, evidence from the oldest terrestrial zircons also suggests that circumstances for stable crust with liquid water were already favored after about 4.4 Ga, or within about 100 Myr or so of the origin of the Moon^19–21^.

Previously, Benner et al.^22^ proposed an under-constrained timeline (4.36 ± 0.1 Ga) for the surface to become hospitable for RNA formation based on assumptions about the cooling time of the crust after large impacts, and the evolution of post-impact transient H_2_-rich atmospheres (cf. Genda et al.^23^). A subsequent analysis^24^ reached a similar central age (~4.35 Ga) but with broader uncertainty (approximately 3.9-4.45 Ga) and a stronger emphasis on the requirement for a highly reducing, hydrogen-rich atmosphere. Others have posited an RNA World at later times, towards the end of the Hadean eon at about 4 Ga^1^. Going beyond these various estimates, we may expect that a swiftly declining impact flux^25^ and a steadily cooling mantle^18^ allowed for the first life forms to emerge early on. For example, evidence shows that dry land existed on Hadean Earth^26^ where prebiotic molecules could be brought to concentration and react^27^. Besides evidence from isotopic and trace element geochemical data that Hadean crust interacted with liquid water and had a significant inventory of continental affinity^28^, a very few of the oldest zircons also host inclusions of isotopically light carbon hinting at a biosphere before 4.10 Ga^29^, although this interpretation remains debated. Other carbon isotopic evidence points to a fully operative biosphere by 3.83 Ga^30^. Molecular clock studies of extant genomes show that the Last Universal Common Ancestor (LUCA) of the contemporary DNA World was already in place by 4.09–4.33 Ga^31^. Evidently, the length of time it took to achieve life on our planet was relatively short and predates the rock record.

Here, we test the hypothesis that surface cooling was rapid enough so that within 150 Myr of the Moon forming event the chemistry leading to the RNA World – a precursor stage in the history of early life before DNA – could endure on Hadean Earth. We perform numerical simulations of post-accretionary bombardment to the crust using the Hadean zircon record as a benchmark, as well as by incorporating estimates of thermal gradients, computed volumes of crust where biomolecule stability is preserved, and track the generation of hydrothermal clusters in the near-surface zone that could have supported a primordial biome. We assess the implications of the timing of the RNA World in light of recent estimates for the appearance the LUCA population.

Any proposed scenario for the origin of life must contend with the fact that impact bombardment is a natural consequence of the planet formation process^32^. Late accretion refers to an extended period of impact bombardment that occurred after the primary stages of planet formation, and after the Moon^33^. In brief, following the formation of planetary cores and the differentiation of silicate reservoirs (i.e., crust and mantle), residual comets, asteroids and leftover planetesimals with intersecting orbits continued to be captured and integrated into terrestrial bodies, providing substantial mass additions in the early stages while waning in intensity up to the present time^34^. Impact heating from such bombardment leads to localized, regional^15,35,36^, or in extreme cases, wholesale melting of the crust^23^. Less intense impacts create hydrothermal oases favorable for life^37^. Whilst late accretionary bombardment in the first billion years of solar system history strongly affected the inner planets, direct lines of evidence for these on Earth are lacking. A thermal history of impact can be preserved in zircon in certain cases such as on the Moon^38^. The oldest known terrestrial zircons have ages up to ca. 4.4 Ga^21^ but it remains unclear whether these host interpretable signatures of impact events (c.f. Trail et al.^39^).

## Results

**Fig. 1:**
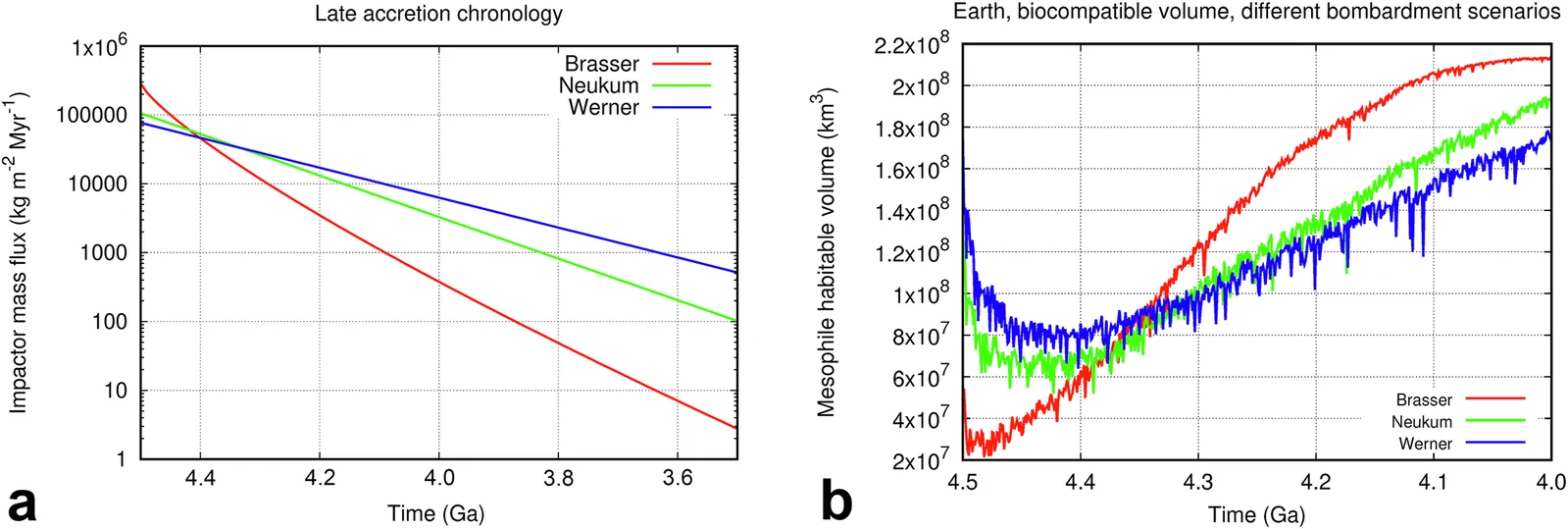
Late accretion mass-production functions. **a** Timeline of late accretion chronologies modeled in this work. The Brasser chronology is a stretched exponential exp[-(*t*/*τ*)^*β*^] with *τ* = 47 Myr and *β* = 0.8. Neukum chronology is an exponential decay with an e-folding time of 144 Myr; Werner chronology is an exponential decay with an e-folding time of 200 Myr. The chronologies are normalized to generate a total delivered mass between 4500 and 3500 Ma of 7.76 × 10^21^ kg, or 0.13 wt%. **b** Total biocompatible volumes for biomolecules under mild temperature (20–50 °C) predicted by the Brasser, Neukum, and Werner impact chronologies. Baseline (pre-formed crust) simulation, total delivered mass is 0.57 wt%.

To explore this further, we use an established three-dimensional bombardment model to assess the global thermal effects of late accretion on Earth’s crust in its first billion years. We couple the results to models of diffusive loss and U-Pb age-resetting in zircon to derive predicted zircon ages. The model uses the Brasser chronology^8,9^ with a conservative high-end delivered mass of 0.57 wt % Earth as the baseline. Additional chronologies^40,41^ and delivered masses are also tested (Fig. 1). The main focus of our analysis is to predict lithospheric melting and subsurface biocompatible volumes and thereby to compute the optimal time for the onset of the RNA World.

To benchmark our results, we compare our predicted impact-induced zircon geochronology to published age spectra of Hadean zircons from the Jack Hills in Western Australia^42^. The comparison is deliberately restricted to the Jack Hills dataset because it remains the largest, oldest, and most extensively studied collection of Hadean zircons. This makes it the natural benchmark for evaluating model predictions against the empirical Hadean zircon record. We note, however, that detrital zircon age distributions within the Jack Hills succession are heterogeneous and vary substantially between different stratigraphic units^43^. Our model-data comparison therefore focuses specifically on the oldest (Hadean) zircon population, in which a prominent ~4.0–4.1 Ga age peak is consistently observed across independent studies^42–44^.

Although the three-dimensional thermal bombardment framework used here builds on earlier work, the present study differs in both scope and scientific objectives from previous applications of the model. Specifically, Abramov and Mojzsis^15^ and Abramov et al.^35^ examined only the thermal effects of a putative Late Heavy Bombardment interval near 3.9 Ga; the former focused on the survival of already-existing microbial life, whereas the latter contained no biological or habitability analysis. In contrast, the work presented herein spans the entire late-accretion period (4.5–3.5 Ga) and provides a quantitative timeline for the emergence of the RNA World. Methodological advances include an overhauled treatment of impact-induced zircon age-resetting, a ten-fold longer simulation duration (1000 Myr versus 100 Myr), a time-varying impactor flux, and variable impact velocities. These improvements are coupled to molecule-specific thermal-stability thresholds for RNA, DNA and RNA-DNA complexes, quantification of never-sterilized crustal volumes, statistical identification of hydrothermal clusters via DBSCAN, and direct comparison with the latest molecular-clock estimates for LUCA.

### Wholesale thermal effects

**Fig. 2:**
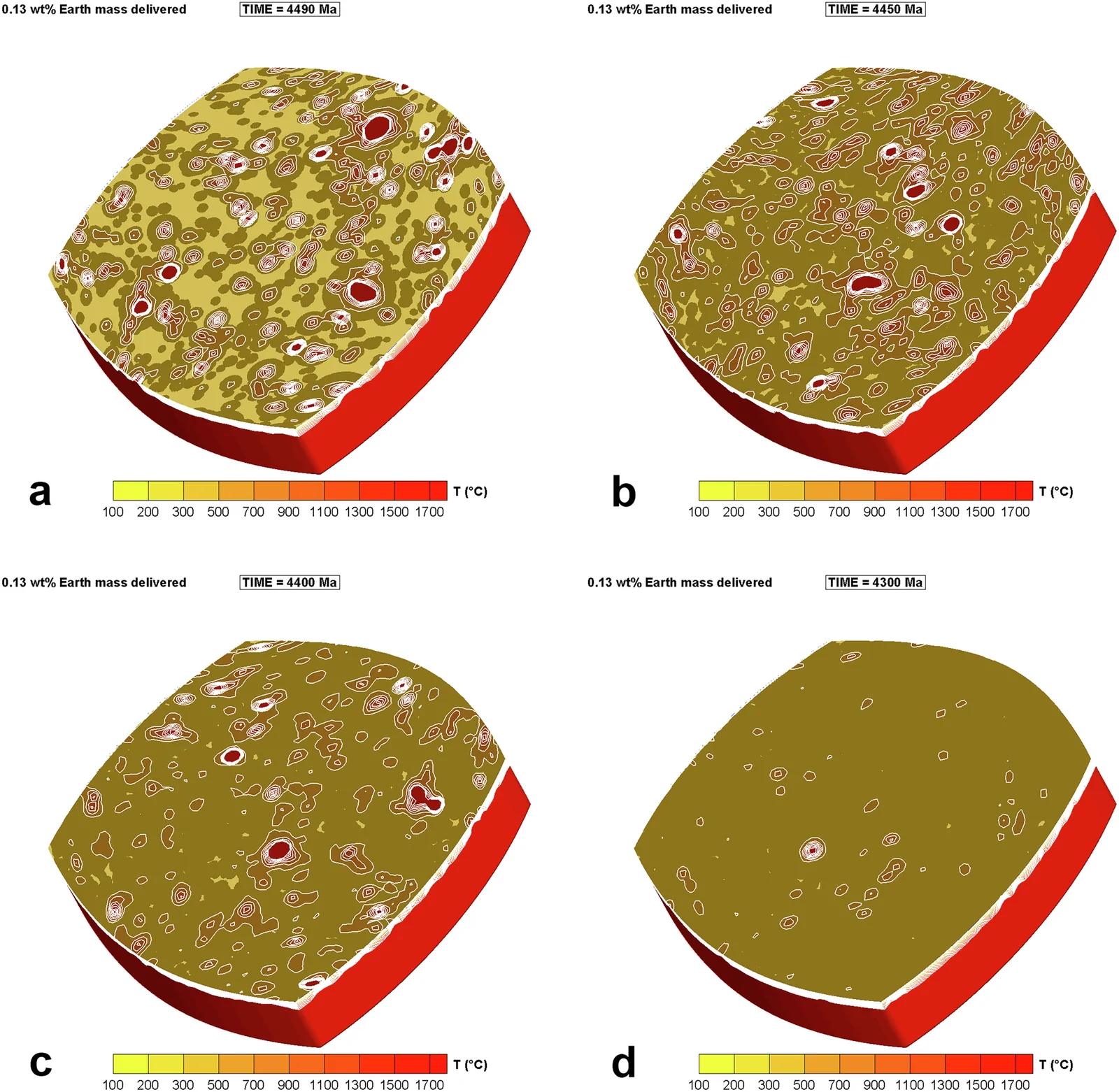
Thermal modifications to the Hadean crust from impact bombardment. A three-dimensional thermal model with 0.13 wt% delivered mass, representing the upper 140 km of the Earth’s subsurface during late accretion. In this simulation, the Brasser impact chronology is used. Dark circles indicate crater locations and approximate final diameters; these symbols are overlaid on the temperature field and do not correspond to temperature values. Note the progressive saturation of the surface by impact craters in later panels. The upper surfaces show temperatures at a depth of ~4 km. **a** 10 Myr into late accretion. **b** 50 Myr into late accretion. **c** 100 Myr into late accretion. **d** 200 Myr into late accretion.

Figure 2 provides a visual representation of typical output of our three-dimensional global model, which depicts the thermal effects of post-accretionary bombardment on the Earth’s crust and upper mantle. This output serves as a basis for subsequent analyses reported herein, including zircon age-resetting via Pb-loss, crustal melting, and biocompatible volumes. In the scenario represented by Fig. 2 the total delivered mass is 0.13 wt%, the smallest tested, but the cratering density nonetheless approaches saturation. This is indicated in the plot by dark circles approximating final rim-to-rim diameters of the resultant impact craters. As in prior work^15^, the visualization is a snapshot of temperatures taken at a depth of ~4 km rather than at the surface. This choice is driven by the rapid radiative cooling that exists at the model’s upper boundary, which prevents meaningful representation of the cumulative effects of impact-induced heating. Note that, although the model shows a relatively large number of craters formed by 4.49 Ga, most of these structures have rapidly cooled below 100 °C even to a depth of ~4 km at that time. It should also be noted that the rapidly diminishing impactor flux, as shown in Fig. 1a, leads to far fewer new craters and thus much less intense impact-induced heating of the subsurface already by 4.3 Ga.

### Computed crustal age distribution from late accretion

We analysed the distribution of the crustal ages predicted by applying equations for Pb diffusion in zircon to the output from our three-dimensional thermal model (Supplementary online material Figure S1). It should be emphasized that these model ages only apply to impact processes as simulated in our work and do not account for normal magmatic processes that generate zircon-saturated magmas. In other words, the synthetic U-Pb age spectra represent only zircon crystallization or Pb-loss resetting induced by impact heating; they deliberately exclude background magmatic zircon formation arising from intrinsic crustal processes such as mantle melting or intracrustal melting. Consequently, our model spectra constitute an “impact-only” end-member. The zircon ages provide an independent benchmark of the thermal histories generated by the bombardment model: if the model had produced synthetic ages substantially younger than the observed Hadean zircon record, it would indicate a problem with the calculated temperature fields.

Interestingly, the presence or absence of an initial global magma ocean in the simulation does not significantly affect the zircon age results owing to the rapid formation of the crust. Also, as the impact flux decreases with time this difference in initial conditions (magma ocean or no magma ocean) becomes negligible for the purposes of thermal modeling by ~about 3.5 Ga. Figure S1a approximates Earth’s Hadean zircon age distribution if no effective crustal recycling existed on our planet (e.g. stagnant lid regime, with no plate tectonics or subduction), and impacts were the only process to affect the age of the crust. If the Earth did not have the ability to recycle, the majority of its upper crust would be very ancient (Figure S1c). In this unrealistic model, the mean age of the crust shifts younger as the delivered mass is increased to 0.57 wt% (Figure S1b,d) thanks to higher net impact fluxes later in the bombardment as compared to the less catastrophic 0.13 wt% case. In the 0.57 wt% test case we find that a significant >4.4 Ga age component still remains in the simulation. Although not relevant to Earth (or Venus) which has had active crustal processing for most if not all of its history, our analysis has implications for planets with moribund dynamics such as Mercury or Mars. For example, our bombardment results comport with the documented presence of martian meteoritic zircons as old as 4.47 Ga^45^.

### Predicted zircon ages, crustal melting, and biocompatibility calculations

**Fig. 3:**
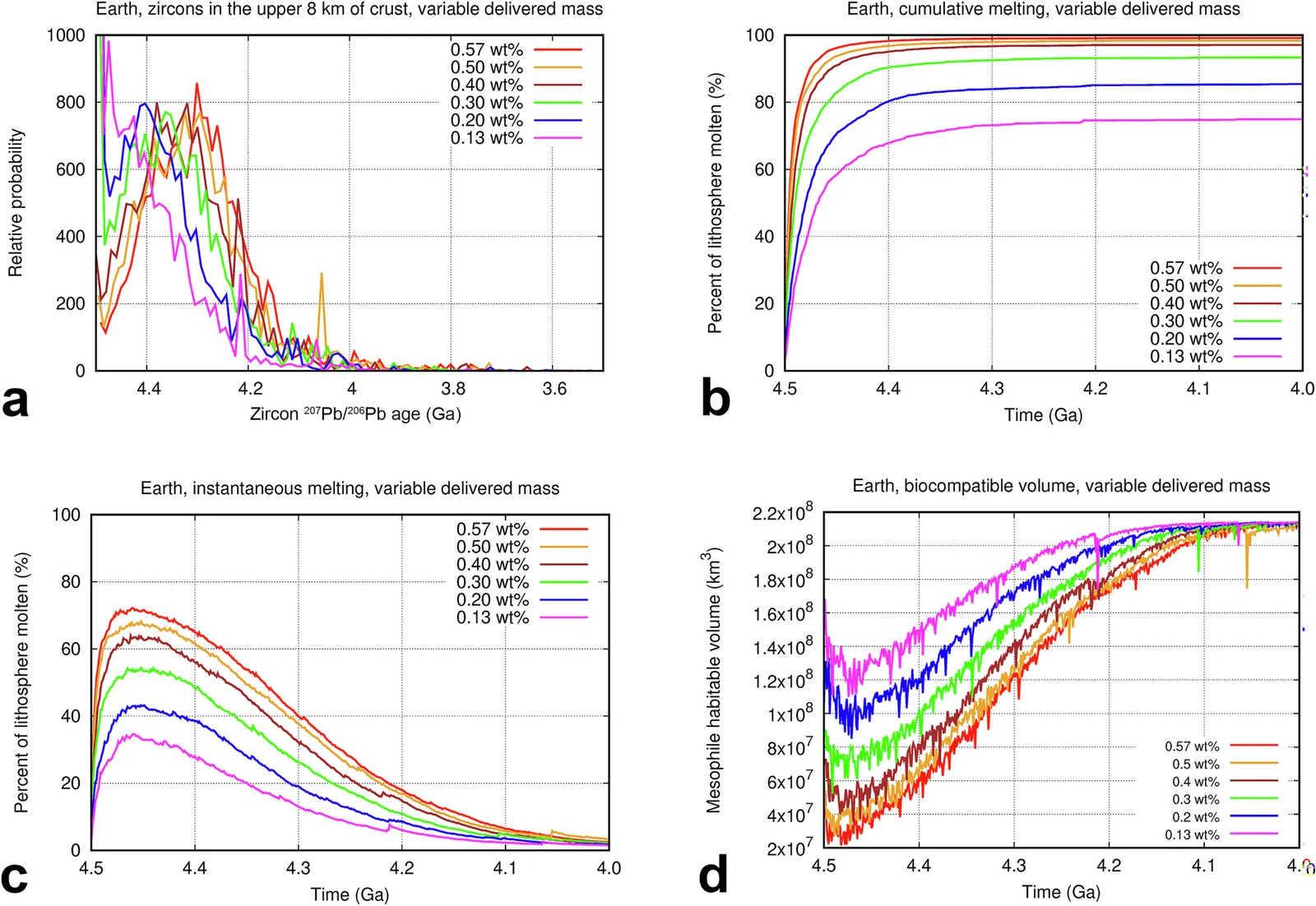
Consequences of late accretion to the Hadean crust. Reported model runs with 0.13, 0.20, 0.30, 0.40, 0.50, and 0.57 wt% delivered mass: **a** Distribution of zircon ages in the upper 8 km of the crust. **b** Percent of lithosphere cumulatively molten. **c** Percent of lithosphere molten at any given time. **d** Volume for biocompatible mild temperatures in the range 20–50 °C.

Figure 3a shows zircon ages predicted by our model for the upper 8 km of the crust for different delivered masses due to thermal effects of impacts on a stagnant-lid crust with no plate tectonics-like recycling. For such a crust, we predict that a large number of very old zircons should exist, peaking in abundance between ~4.3 Ga and ~4.5 Ga. Higher delivered masses result in younger age peaks. Figure 3b shows cumulative melting of the lithosphere, calculated by adding up lithospheric volumes that undergo melting at any time between 4.5 and 3.5 Ga due to impacts. For the 0.13 wt% delivered mass, ~75% of the crust experiences melting over the duration of the bombardment. For 0.57 wt% delivered mass, this number reaches almost 100%. Instantaneous melting (Fig. 3c), meaning the fraction of the lithosphere molten at any particular time during bombardment, varies from ~30 to ~70% depending on delivered mass. Impact-induced melting peaks at ~4.47 Ga independent of delivered mass.

In Fig. 3d we have adjusted the thermal parameters of the model to map a volume for the upper crust defined by mild temperatures <20–50 °C consistent with the preservation of biomolecules (Supplementary online material Table S2). Our result shows that this volume reaches a minimum at ~4.47 Ga coinciding with the maximum instantaneous melting of the crust at that time (see Fig. 3c). Afterwards, the computed biocompatible volume of the Hadean crust increases with time as the bombardment decreases in intensity, thus allowing the near-surface to sustain cooler temperatures.

Next, we explore the instantaneous lithospheric melt fraction over time for a pre-formed crust (baseline model) and an initial global magma ocean condition (Supplementary online material Figure S2). The pre-formed crust case begins with 0% instantaneous melt at 4.5 Ga, which afterwards rapidly increases owing to impact-induced heating of the crust. This value reaches a peak of ~70% at ca. 4.47 Ga. Subsequently, we find that the melt fraction decreases with decreasing impact rate. In contrast, the initial magma ocean case starts with 100% melt at 4.5 Ga and decreases owing to crust formation. The melt fraction in the magma ocean scenario is at first significantly higher than for the pre-formed crust condition, highlighting how the remnant gravitational heat from planet formation is magnified by bombardment. However, by ~4.4 Ga the two scenarios (pre-formed crust and magma ocean) diverge by less than 5%. This suggests that the crust is fully formed by that time in agreement with the documented Hadean zircon record^19^. To study this further, we compare the zircon U-Pb ages predicted by our model for the upper 8 km of the crust using the Brasser, Neukum, and Werner post-accretionary bombardment chronologies (Supplementary online material Figure S3a). The delivered mass in this model is 0.57 wt%, the highest tested in this analysis. However, even in this scenario, the predicted peaks for synthetic U-Pb zircon ages (~4.15 to ~4.3 Ga) are markedly older than the observed age distribution of Hadean zircons (Supplementary online material Figure S3b). This implies that additional non-impact processes were required to generate the younger zircons that dominate the natural Hadean record.

### Volumes of near-surface biocompatibility and diversity of hydrothermal systems

**Fig. 4:**
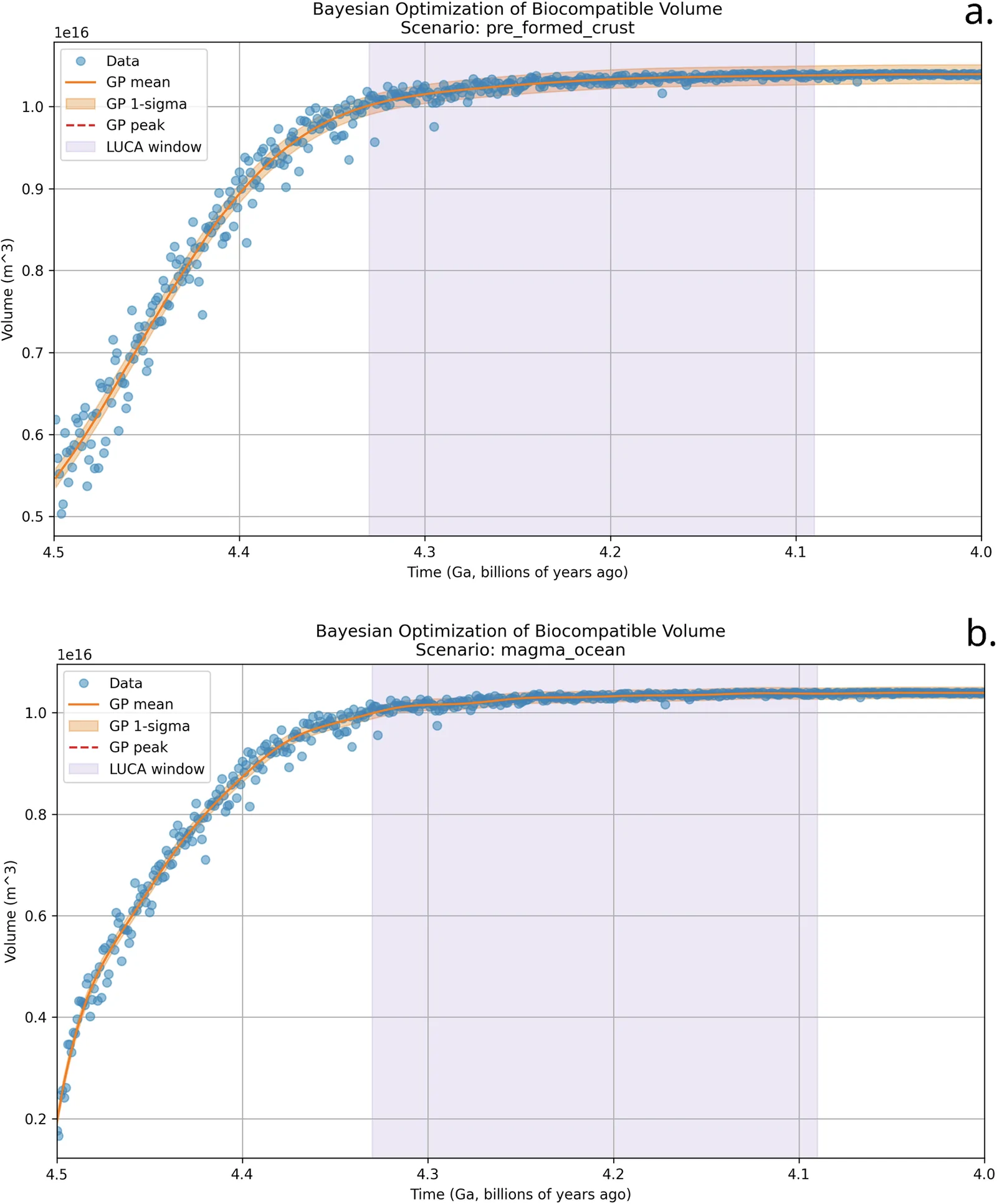
Biocompatibility estimates. Biocompatible volume (<110 °C) in the upper 300 m of the Hadean crust for the (**a**) baseline pre-formed crust scenario and (**b**) magma ocean scenario. Gaussian process (GP) regression fits with confidence intervals (CI) are presented. The LUCA molecular clock window (4.09-4.33 Ga) is shown in purple. Total delivered mass is 0.57 wt%, volumes are scaled by 5% land exposure.

The near-subsurface (upper 300 m) of biocompatible volume defined by temperatures <110 °C and scaled to 5% land exposure is illustrated in Fig. 4. All scenarios show global conditions that militate biomolecule stability until ca. 4.45 Ga, followed by a rapid rise to >90% of the volume of the near-subsurface supporting mild conditions by 4.3 Ga. Gaussian process regression reveals flattening temperature curves after 4.4 Ga which we interpret to mean that impacts no longer dominate global thermal regimes. Overlain on this output is the window for the emergence of the LUCA as reported from analysis of molecular clocks (4.09–4.33 Ga)^31^. The proximity of biocompatible conditions and the LUCA suggest to us that the RNA World emerged shortly after the flattening of the biocompatibility curve in Fig. 4. The magma ocean scenario initially hosts much lower biocompatible volumes, but, as in the case with lithospheric melting, diverges by less than 5% from the pre-formed crust scenario by 4.4 Ga. Thus, independent of the impactor mass flux or the initial crustal scenario, the answer remains the same; conditions for the emergence of life on Earth were established after 4.4 billion years ago.

**Fig. 5:**
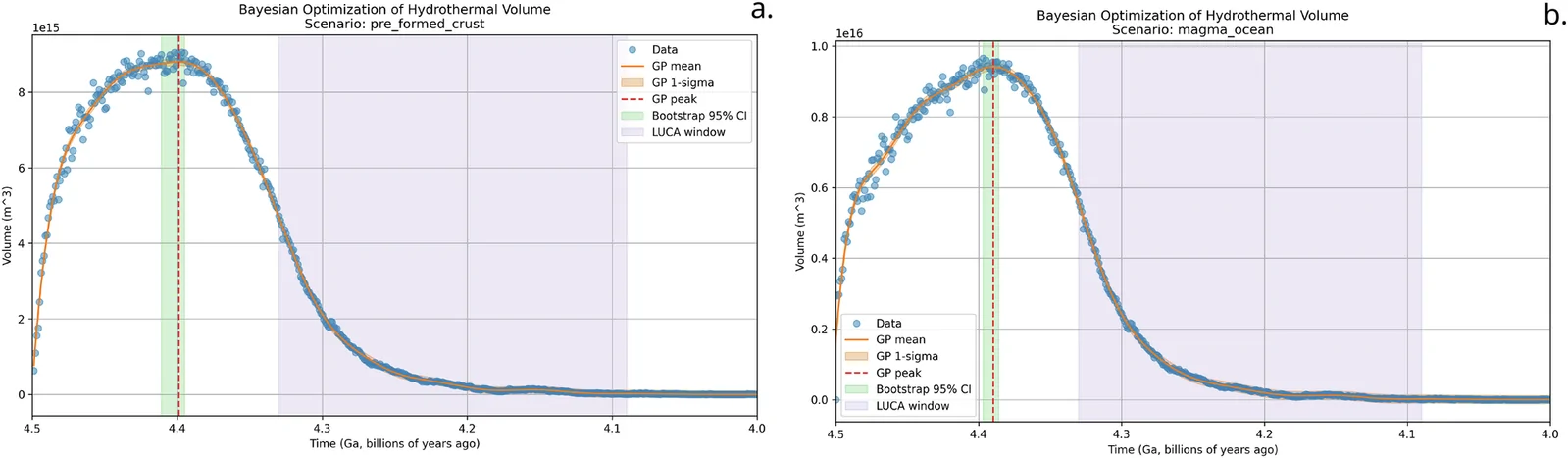
Appraisal of hydrothermal volumes. Hydrothermal volume (<110 °C and > 140 °C/km thermal gradients) in the upper 300 m of the Hadean crust for the (**a**) baseline pre-formed crust scenario and (**b**) magma ocean scenario. Other parameters are the same as that reported in Fig. 4.

Turning now to the habitats that could serve as the crucible for an RNA World, Fig. 5 quantifies hydrothermal zones (i.e. high geothermal gradients >140 °C km^-1^, temperatures fields <110 °C) within the upper 300 m of the crust. Our results show that hydrothermal volumes defined by these parameters peak at ~4.4 Ga and afterwards show a decline to ~20% of the maximum by 4.3 Ga. This is because by this time bombardment has abated dramatically and thereafter decreases more gradually following the evolution of the impactor flux. It is interesting to note that the data spread narrows post-peak, which we interpret to reflect stabilizing thermal conditions. Furthermore, the difference in peak location between the pre-formed crust and initial magma ocean scenarios is minimal. We now focus our analysis only on the pre-formed crust scenario post 4.4 Ga.

Although the global three-dimensional model used here does not explicitly simulate hydrothermal fluid circulation, hydrothermal activity can significantly affect the temperature of the near-surface. Detailed numerical models of individual impact craters^37,46^ show that the effect depends strongly on crustal permeability. At low permeabilities little heat is transported by flowing water to the near-surface, resulting in lifetimes similar to the purely conductive case. As permeability increases, significant amounts of heat are delivered to the near-surface, lengthening the lifetime of the hydrothermal system. At still higher permeabilities this effect is negated by the overall rapid cooling of the system, so that lifetimes eventually fall below those of a purely conductive case. In other words, hydrothermal circulation can either prolong or shorten elevated near-surface temperatures depending on local permeability. Recent analysis of the Chicxulub hydrothermal system^47^ supports these earlier findings by demonstrating that parts of the near-surface remained warmer for longer than would be expected from pure conduction alone. Consequently, the temperature-based hydrothermal volumes reported in the present study should be interpreted as a first-order, conservative indicator of regions capable of hosting long-lived, chemically active environments after large impacts.

### Parameterization of near-subsurface temperatures and thermal gradients

**Fig. 6:**
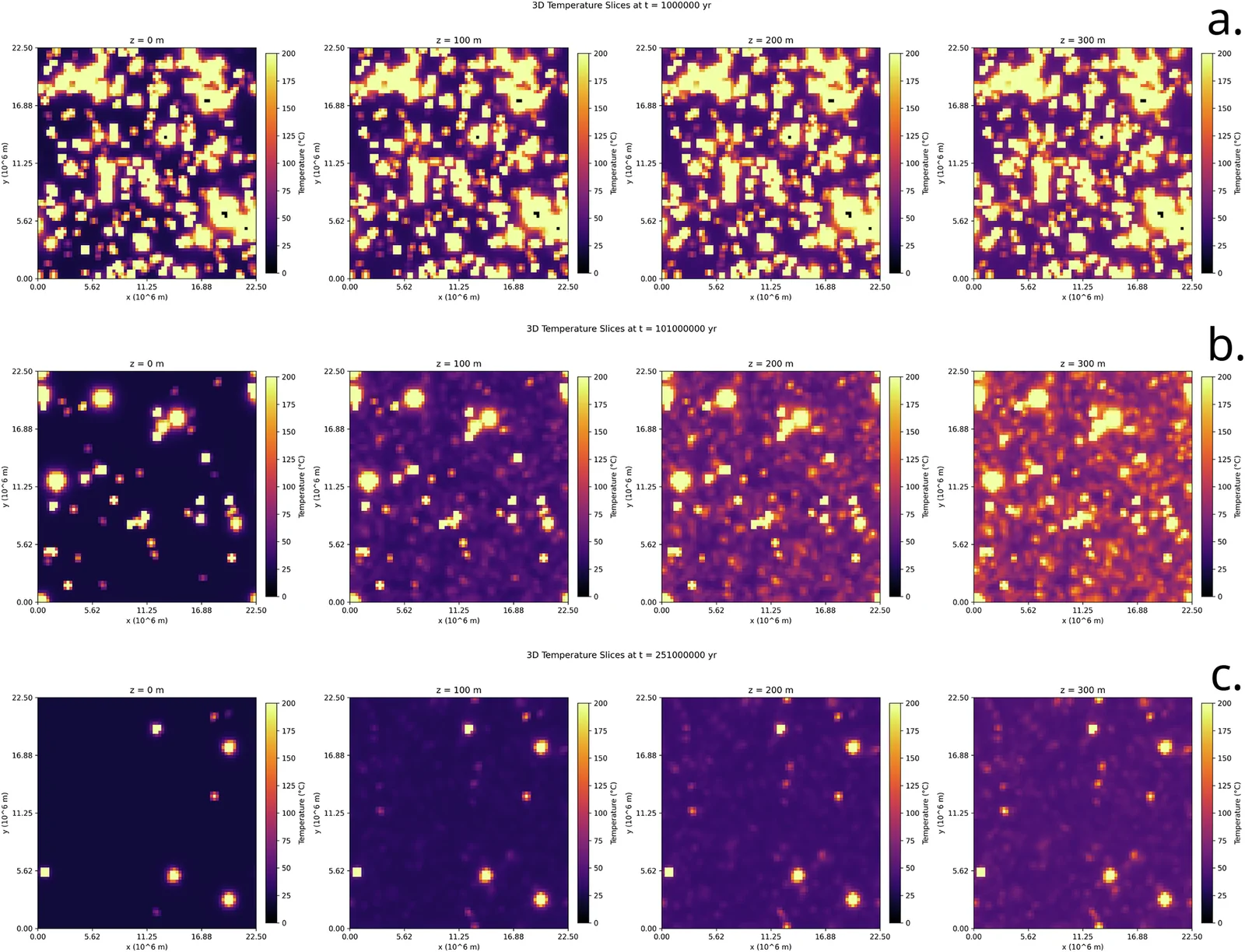
Shallow sub-surface thermal regimes. Temperature slices in the Hadean subsurface at depths of 0, 100 m, 200 m, and 300 m at: (**a**) 1 Myr (4.5 Ga), (**b**) 101 Myr (4.4 Ga), and (**c**) 251 Myr (4.25 Ga). Baseline (pre-formed crust) simulation, total delivered mass is 0.57 wt%.

Next, we created horizontal temperature maps at different depth slices and time points (Fig. 6). As can be seen at 4.5 Ga (1 Myr post-start) temperatures exceed 200 °C over at least 50% of the Hadean surface globally thanks to intense impact heating. These temperatures likewise increase with depth following the geothermal gradient. Our model does not explicitly incorporate heat from impact ejecta and condensed rock vapour, which would further increase temperatures to >200 °C for the entire system (100% of the surface) at that time. By 4.4 Ga, it can be seen that the surface presents several hotspots due to recent impacts and thermal remnants of older impacts that become more apparent with increasing depth. Already, we find that some habitable niches begin to emerge even when the effects of hot ejecta are considered. Finally, by 4.25 Ga, it is apparent that profiles stabilize near geothermal gradients (70 °C km^-1^), which is an indication of significantly reduced impact heating and instead extensive mild (<20–50 °C) volumes.

**Fig. 7:**
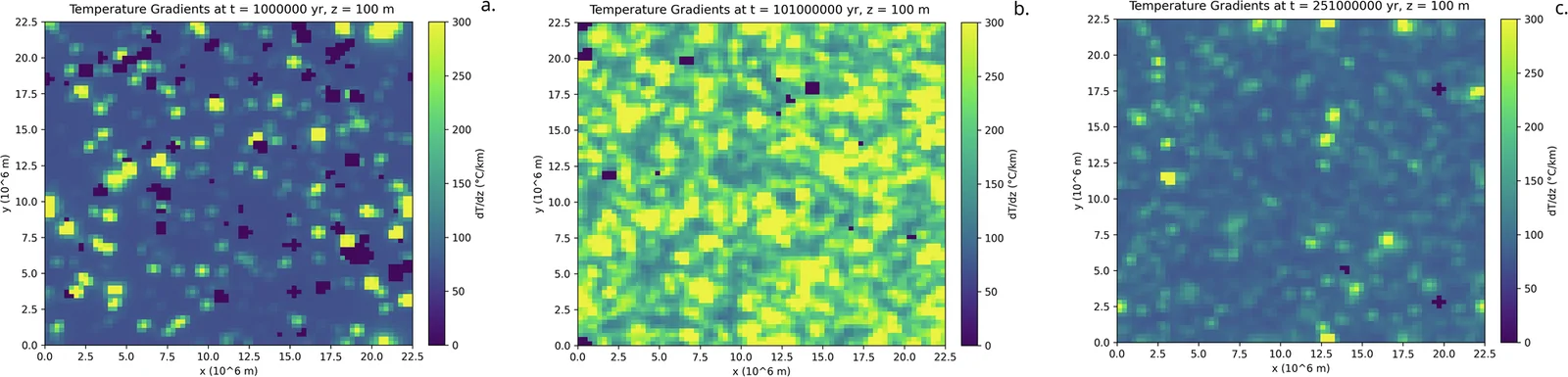
Temperature profiles. Vertical temperature gradients at a depth of 100 m in the Hadean crust at (**a**) 1 Myr (4.5 Ga), **b** 101 Myr (4.4 Ga), and (**c**) 251 Myr (4.25 Ga). Baseline (pre-formed crust) simulation, total delivered mass is 0.57 wt%.

**Fig. 8:**
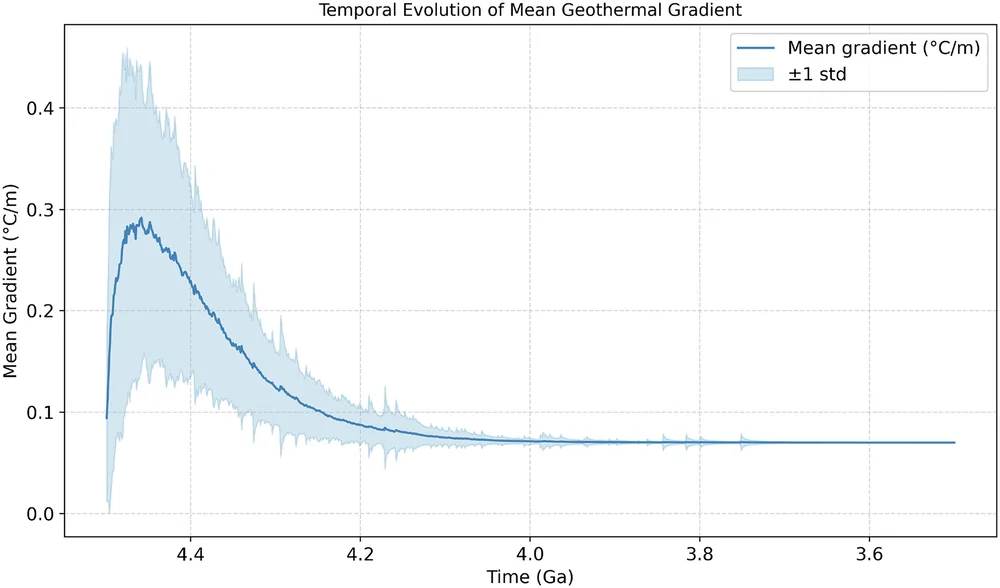
Geothermal gradient evolution. Temporal evolution of the mean geothermal gradient of the Hadean and Eoarchean crust and its standard deviation (std) over 1 Gyr of simulation time (0 Gyr = 4.5 Ga) in the upper 4 km of the crust. Baseline (pre-formed crust) simulation, total delivered mass is 0.57 wt%.

Turning our attention to yet shallower depths for biocompatibility in the sub-surface, Fig. 7 maps vertical thermal gradients at 100 m revealing high variability and numerous islands of high geothermal gradients as impacts begin to heat the pre-formed crust at 4.5 Ga. We find that by 100 Myr after the start of the simulation, the entire crust has been thoroughly heated by impacts, resulting in globally elevated geothermal gradients. As expected, by 4.25 Ga the gradients subside with the exception of a few hotspots associated with recent impacts. This progression is summarized in Fig. 8, which maps the evolution of the mean geothermal gradient in the upper 4 km of the Hadean crust with time. Data show that there is a peak in geothermal gradient of almost 300 °C km^-1^ at ca. 4.47 Ga induced by cratering, thereafter moderating to ~210 °C km^-1^ by 4.4 Ga, then decreasing to ~120 °C km^-1^ by 4.3 Ga, and finally approaching the baseline value of 70 °C km^-1^ by ~4.1 Ga. This thermal evolution of the early Hadean crust underscores the transition from an impact-dominated to conduction-dominated crustal thermal regime at 4.33 Ga, when the geothermal gradient becomes less than twice its equilibrium value.

### Instantaneous biocompatible volumes

**Fig. 9:**
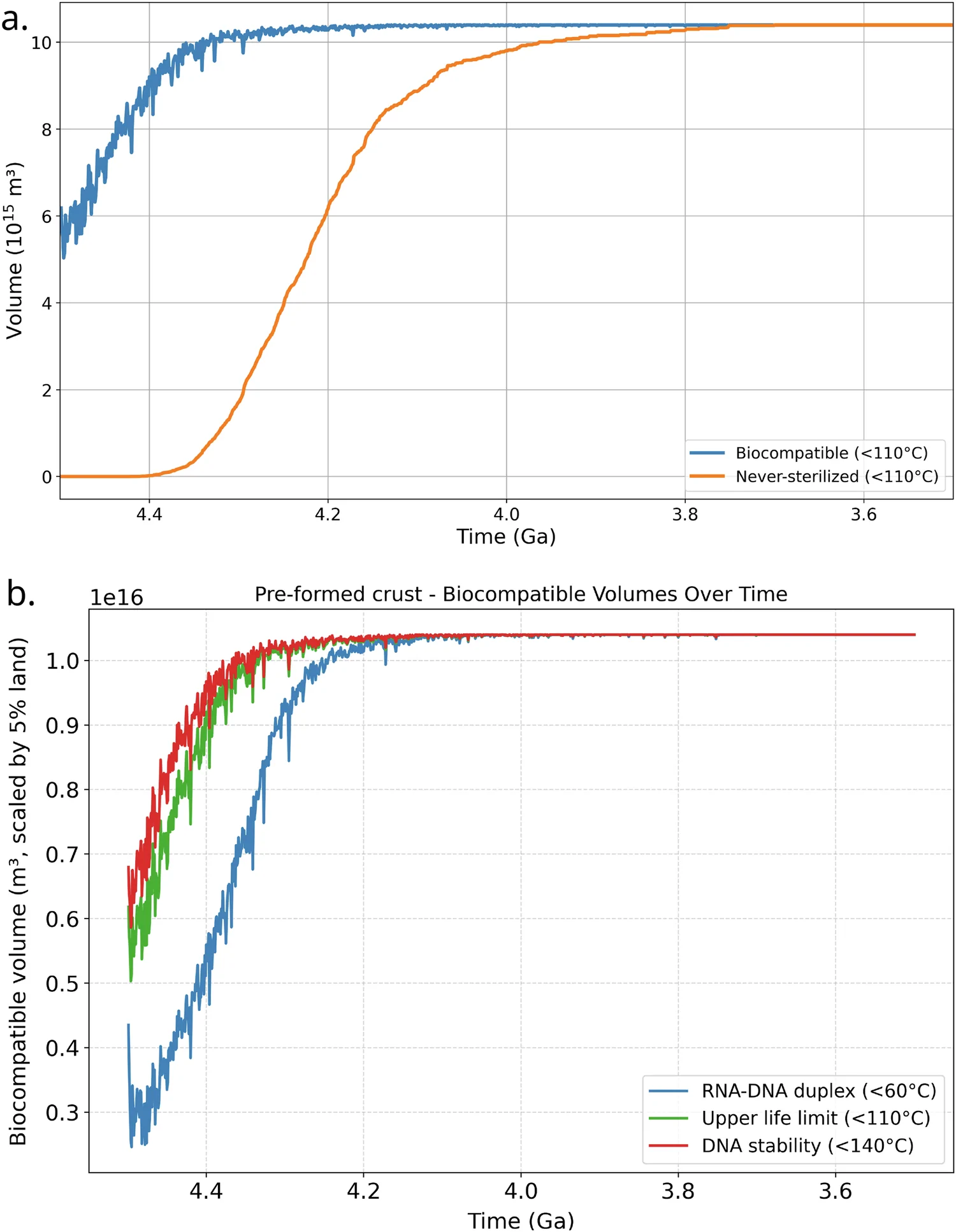
Persistent biocompatibility. **a** Time series of *never-sterilized volume* (volume that never exceeds 110 °C after a time specified on the *x*-axis) in the upper 300 m of the Hadean crust. Biocompatible volume (volume at temperatures below 110 °C) is shown for comparison. **b** Time series of volumes for DNA stability (<140 °C) and RNA-DNA complex stability (<60 °C) in the upper 300 m of the crust. Baseline (pre-formed crust) simulation, total delivered mass is 0.57 wt%, volumes are scaled by 5% land exposure.

In our simulations, we now compare *never*-sterilized volumes in the upper 300 m of the Hadean crust–defined as those regions that at no subsequent time exceed 110 °C after initial cooling–to the *instantaneous biocompatible volume* over the same period of time. As shown in Fig. 9a, we find that the never-sterilized volume of the Hadean crust only exceeds non-zero values after ~4.4 Ga. This result establishes the time when the first mild temperature “sweet spots” existed which prevented thermal degradation of biomolecules or sterilization of incipient lifeforms in the Hadean crust. This volume grows with time to exceed 50% of the crust by 4.25 Ga. In Fig. 9b we report our analysis comparing both the thermal stabilities of biomolecules (RNA only, and RNA-DNA duplexes) and upper temperature limit of hyperthermophilic microbes in the upper 300 m of the crust over time (see Supplementary online material Table S2). We take ≤60 °C as a conservative threshold for maintaining overall biomolecule stability providing for the origin of the RNA World. By tracking the ≤60 °C temperature parameter our model reveals that the volume of crust mild enough to preserve the building blocks of an RNA World begins to exceed 50% of the equilibrium baseline only after 4.4 Ga.

**Fig. 10:**
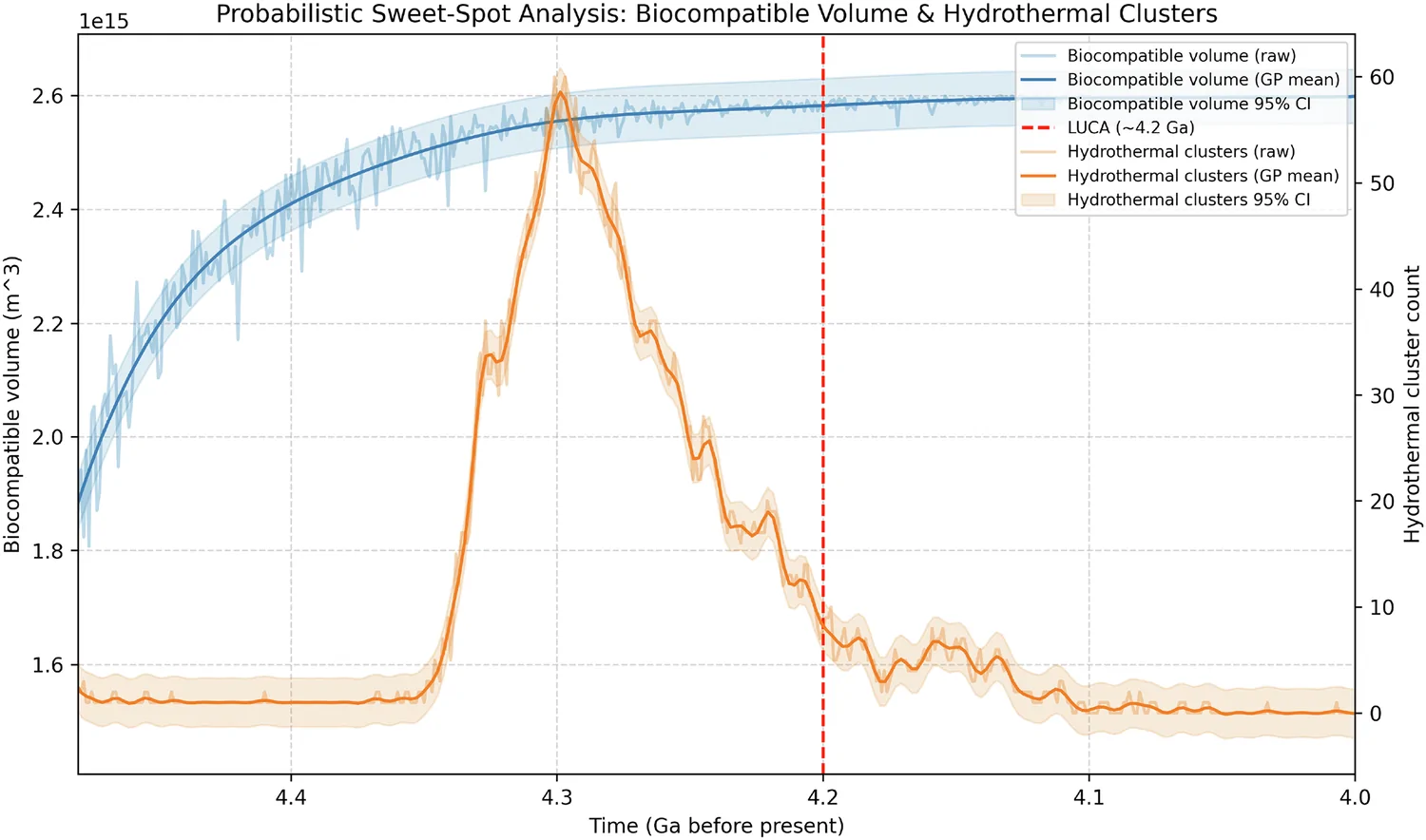
Clustering of Hadean hydrothermal systems. Number of hydrothermal clusters (non-interconnected hydrothermal zones) with time. Biocompatible volume in the upper 100 m of the Hadean crust is shown for comparison. Gaussian process (GP) regression fits with 95% confidence intervals (CI) are presented. Baseline (pre-formed crust) simulation, total delivered mass is 0.57 wt%, volume is scaled by 5% land exposure.

Figure 10 reports the results of our analysis of the number of impact-induced hydrothermal clusters on Hadean Earth as potential sites for prebiotic chemistry and subsequent RNA World emergence. We define such clusters as being composed of individual interconnected hydrothermal systems, and output shows that the global abundance of these habitats rapidly peaks at 4.3 Ga and thereafter gradually declines.

### Vita interrupta

We were interested in understanding the risk that the inception of an RNA World on Hadean Earth could be cut short by one or more late sterilizing impact events. To investigate this, we first compiled a chronological distribution of large impacts capable of sterilizing global oceans, binned in 10 Myr intervals from 4.5 to 4.29 Ga (Supplementary online materials Table S3). As expected, the highest likelihood for sterilizing impacts – defined as ocean vapourizing events–occurs early in the simulation with 6-7 such impacts possible per bin from 4.5–4.48 Ga. After 4.43 Ga, the threat declines to rare sporadic events (potential for 0–1 per bin). This pattern is explained by the rapid decay in impactor flux under the Brasser chronology which predicts a probability for ~40 such global sterilizing events in the time span 4.5–4.29 Ga. As previously mentioned, sensitivity tests performed with both the Neukum or Werner chronologies do not change this outcome.

Our model further shows that near-surface temperatures in volumes of the crust after 4.43 Ga did not necessarily exceed values destructive to life or to organic precursors of life at all places all at once (Supplementary online material Table S2). This is an important distinction, because patches of Hadean Earth’s crust could be conducive to prebiotic chemistry for a time, only to later be destroyed by local (small) or regional (large) impact events and associated thermal assaults. For example, we find that even at ca. 4.45 Ga some temperatures in near-surface (100-300 m depth) volumes unaffected by recent impacts are below 50 °C (Fig. 6b). Concurrently, impact-produced heat resulted in localized temperatures exceeding 200 °C at 300 m depth to produce some very high geothermal gradients (Fig. 7b, Fig. 8) that drive intense hydrothermal systems (Fig. 5). The water in such systems reached temperatures ranging from moderately warm to well above 100 °C, as observed in modern terrestrial hydrothermal vents^48^. By the time of the LUCA at an average of about 4.2 Ga, however, impact-induced temperatures were around 25 °C to 50 °C at a depth of 300 meters (Fig. 6c) and the geothermal gradient was lower (Fig. 7c, Fig. 8). This implies overall milder conditions for hydrothermal activity (Fig. 5) and a world primed for subsequent biological evolution to more complex forms leading to the contemporary domains of life.

## Discussion

To evaluate the sensitivity of these results to the assumed late-accretion mass, we repeated the key biocompatibility calculations with a more modest delivered mass of 0.13 wt%, corresponding to the lower end of dynamical estimates (Figure S4). Under this reduced impactor flux, the criteria for the “sweet spot” shifts earlier by approximately 70 Myr, to ~4.4 Ga. The earlier establishment of favorable conditions would lengthen the available interval for prebiotic chemistry prior to LUCA. Thus, the general conclusion that persistent near-surface niches suitable for the RNA World became established some time after ~4.4 Ga is robust across the full range of delivered masses examined in this study.

At the high end of the delivered mass scale, we adapt 0.57 wt% of Earth’s present mass as a conservative upper-bound scenario consistent with highly siderophile element (HSE) abundances in the mantle under the assumption of efficient metal retention. Recent SPH impact simulations^49^ indicate that metal retention would be incomplete for large (≥1,500 km diameter), differentiated projectiles, suggesting the potential for even larger delivered mass. In contrast, the size-frequency distribution used in the present work is based on the main asteroid belt and, when combined with the same HSE mass constraints, yields impactors smaller than ~700 km and therefore mostly undifferentiated.

However, delivered mass remains a critical and uncertain parameter. Extrapolating the results for the 0.13 wt% delivered mass described above, a further doubling of the delivered mass beyond 0.57 wt% would be expected to shift the results by no more than a few tens of millions of years toward younger ages (for example, to ~4.30 Ga rather than 4.33 Ga).

Whilst our primary analysis has focused on the thermal conditions required for the persistence of an RNA World, the results have broader relevance for alternative origin-of-life scenarios. Metabolism-first models^50,51^, for example, propose that self-sustaining chemical reaction networks (powered by geothermal energy, mineral catalysts, and proton gradients rather than sunlight) preceded or evolved in parallel with the emergence of genetic information-storage molecules. The energy sources invoked in these metabolic models are directly analogous to the impact-generated hydrothermal oases that our simulations indicate became persistent and widespread after ~4.4 Ga. Although the upper temperature thresholds for the stability of many organic compounds and metabolic intermediates appear broadly comparable across these frameworks, the efficiency and specific pathways of the underlying reaction networks are expected to be strongly temperature-dependent. Consequently, the persistent mild-temperature niches rich in chemical disequilibria that emerged after ~4.4 Ga would have provided suitable physical and energetic conditions for either class of model (or for hybrid RNA-peptide scenarios with metabolic capabilities). We note, however, that the quantitative magnitude of temperature effects on prebiotic reaction kinetics remains difficult to constrain with current understanding and therefore lies beyond the scope of the present thermal modeling study.

Additionally, it is important to mention that recent work has proposed an alternative in which RNA-peptide chimeras (or amino-acid-functionalized RNA) were present from the earliest stages of molecular evolution, forming an “RNA-peptide world” rather than an origin from a purely RNA-based system^52^. Coevolution of such chimeric molecules could have facilitated, molecular stability and therefore early adaptation and diversification.

A central chemical challenge for RNA-first scenarios is the need for reduced nitrogen species (primarily ammonia) to form the nucleobases, in contrast to the redox-neutral atmosphere that is thought to have dominated the Hadean. Transient reducing atmospheres generated by large impacts via disruption of metallic cores and subsequent release of H₂ provide a plausible mechanism^23^ for episodically supplying the necessary reduced nitrogen and other reduced species. In this context, Sutherland^1^ has proposed a hypothesis that incorporates heavy impact craters act as intermittent aqueous reservoirs in which rainwater washes out organics from the surrounding regolith and any material delivered by the impactor itself; these reservoirs can repeatedly dry and refill, concentrating prebiotic reactants. Our post-4.4 Ga thermal window coincides with the period when such episodic reducing conditions could have been productively exploited within stable near-surface niches that were no longer subject to frequent global sterilization.

Even though impact-induced hydrothermal systems can generate steep geothermal gradients and chemical disequilibria favorable for early metabolism and mineral catalysis, hydrothermal vents are not required for all actionable models of prebiotic RNA synthesis. Many synthesis pathways, including cyanosulfidic chemistry, can operate in constrained subaerial, intermittently hydrated aquifers under redox-neutral atmospheric conditions. A long-standing alternative hypothesis holds that submarine hydrothermal systems, through their interaction with seawater, create redox gradients and may supply reduced nitrogen via serpentinization; in the presence of transition metals these environments have been proposed to facilitate the abiotic formation of nucleic-acid and amino-acid precursors and even to host chemoautotrophic protocells within mineral compartments^51^. Whether this scenario accurately reflects the origin of life remains debated, and we emphasize that our thermal-stability analysis is deliberately agnostic to the specific setting for abiogenesis. Our maps the physical prerequisite (persistent mild temperatures in the surface and near-surface crust) common to both impact-generated hydrothermal oases and subaerial aquifer models.

The most important outcome of this study is that, based on the average of the criteria described above, we find a temporal “sweet spot” at ~4.33 Ga, representing the maximum biocompatibility potential for the inception of the RNA World. This occurs an average of about 130 Myr prior to the appearance of LUCA at ca. 4.2 Ga, the approximate mean of the 4.09-4.33 LUCA window^31^. Additional geochemical modeling^27^ independently places the prebiotic formation of RNA at ~4.3 Ga. This estimate is fully consistent with the temporal “sweet spot” of ~4.33 Ga identified in the present study and further supports the conclusion that conditions favorable for an RNA World were established shortly after the cessation of frequent sterilizing impacts. We are reminded that LUCA is by definition the *population* of the last universal common ancestor and *does not necessarily represent the first appearance of DNA-based organisms*. This important distinction means that a long history of evolution preceded the LUCA and that significant biodiversity could have existed within the RNA-, Ribonucleoprotein (RNP-) and nascent DNA populations that came before.

Our model results collectively indicate that conditions on the Hadean Earth transitioned from impact-dominated sterilization to persistent near-surface niches optimal for prebiotic chemistry and persistent biochemistry due to extensive hydrothermal activity. Our analysis also shows that potentially ocean-sterilizing impacts (400–500 km diameter) become rare after 4.4 Ga (Table S3), with fewer than 1 per 10 Myr bin. This outcome explains both the preservation of crust and liquid water persistence at that time^19^. It has long been held that early impact bombardments may have generated transient steam atmospheres to raise surface temperatures well above 100 °C from deposition of hot ejecta and rainout of rock vapour; the largest of these had the potential to evaporate and/or sterilize the global ocean^13^. Although our numerical bombardment model does not explicitly include the distal effects of impacts such as hot ejecta deposition and increase in global ocean temperature, previous work on hot ejecta deposition over a water saturated surface suggested that some habitable niches may still remain at depths of a few hundred meters in the subsurface following even some of the largest impacts^15^.

Further, the outcomes of our simulations–compared as they are to the key molecular components of a hypothetic RNA lifeform–contribute to a growing body of evidence that suggests impacts may not have been the primary influence on the age determination of Hadean zircons^35,53^. The model’s predictions, even when considering an unrealistically high delivered mass scenario, consistently yield zircon ages expected from the crystallization of impact melts that are significantly older than those observed empirically from the detrital Hadean zircon record. This discrepancy implies that other geological processes besides impacts were more important to crust formation or destruction. These findings align with other research in the field. For example, most Hadean zircons formed at temperatures significantly lower than what one would expect from impact melt sheets^54^. If impacts were the primary determinant of zircon age, we would expect to see more evidence of high-temperature formation. Moreover, thermobarometry measurements suggest the formation of some Hadean zircons at relatively low temperature, high pressure conditions more consistent with subduction^55^, in contrast with the high-energy, high-temperature typically associated with impacts.

Our global 3-D thermal model used to monitor the stability of the biomolecules that comprise life shows that prior to 4.4 Ga the early Hadean Earth was incapable of hosting persistent biochemistry. It was only afterwards that the bombardment flux and geothermal gradients decreased to that point that the crust could foster hydrothermal niches optimal for life’s emergence. Under conservative high-mass bombardment scenarios, hydrothermal activity peaks at around 4.33 Ga, with local volumes hosting steep geothermal gradients (>140 °C/km) that provided for widespread energy-rich oases that could act as a chemical crucible for the first biosphere. After this time, mean geothermal gradients decline to the point that enables effective and persistent stable preservation of RNA, RNP and DNA structures comprising the core components of the first living cells. In conclusion, we define a temporal “sweet spot” centered around 4.33 Ga for the origin of the RNA World, a timeline that dovetails well with estimates for the appearance of the Last Universal Common Ancestor that subsequently gave rise to all contemporary life on our planet.

## Methods

### Impact bombardment model

The updated impact bombardment model used in this work is built on that described in Abramov and Mojzsis^15,36^ and Abramov et al.^35^. It consists of (i) a stochastic cratering simulation which populates the surface with tens of thousands of craters within specified constraints over a modeled time window expressed in Myr time steps; (ii) analytical expressions that calculate a temperature field for each crater^56,57^; and (iii) a three-dimensional thermal model of the global lithosphere where individual craters are allowed to cool by conduction and radiation. Our analytical approach allows us to perform simulations of thousands of simultaneous impacts covering a solid planet surface that would otherwise be computationally costly with a multi-material, multi-rheology shock physics code (i.e. hydrocode) such as iSALE^58^.

The three-dimensional model is composed of 76 × 76 × 34 nodes, corresponding to horizontal inter-node distance of ~300 km and vertical inter-node distance of ~4 km. Near-surface geothermal gradients and temperatures within 300 m of the surface are calculated by linearly interpolating between the top two nodes of the model. This approximation is valid at the output frequency of the model (1 million years), at which time the upper 4 km temperature-vs-depth curve would be expected to be linear to within ~2%.

Impact melt volumes predicted by this model are consistent well-established analytical scaling relations^59^. Recent hydrocode-based scaling laws^60^ show good agreement, with the earlier results essentially bracketing the newer values.

### Bombardment parameters

We present our thermal impact modeling results for the Earth between 4.5 Ga and 3.5 Ga based on the late accretion bombardment scenario dominated mass-wise by rocky planetesimals^9^. Figure 1a illustrates the impactor mass flux as a function of time for the three chronologies examined in this study and as previously described in Mojzsis et al.^25^: (1) the Brasser chronology^9^, which was used for most of the simulations presented in this paper, and the (2) Neukum^40^, and (3) Werner^41^ chronologies, which were also tested. In the Brasser chronology, the impactor flux as a function of time *t* is described by:

1 $$F\left(t\right)={F}_{0}\,\exp [-{(t/\tau )}^{\beta }]$$

where *τ* = 47 Myr is the characteristic e-folding timescale of impactor flux decay and *β* = 0.8 is a stretching parameter. The value of *τ* reflects the rapid dynamical depletion of the leftover planetesimal population after the main stage of terrestrial-planet formation. The stretching exponent *β* < 1 arises due to a combination of several effects: impacts onto the planets, impacts onto the Sun and ejection from the Solar System. *F*_0_ is a normalization constant fixed so that the time-integrated delivered mass between 4.5 Ga and 3.5 Ga equals a prescribed total (0.13-0.57 wt% of Earth’s present mass).

For the Neukum and Werner chronologies, the impactor flux is given by:

2 $$F\left(t\right)={F}_{0}\exp (-t/\tau )$$

with e-folding times *τ* = 144 Myr and 200 Myr for Neukum and Werner chronologies, respectively.

The three formulations therefore differ in both functional form and characteristic timescale because they rest on different dynamical assumptions and calibration datasets. The Brasser stretched-exponential model, derived from N-body simulations of planetesimal depletion, predicts a steeper early decline, whereas the Neukum and Werner exponential models, tied more closely to the observed lunar crater chronology, decline more slowly. We test all three chronologies to assess the robustness of our biocompatibility results to these alternative descriptions of late-accretion flux.

As can be seen in Fig. 1a, the Brasser chronology predicts a high initial impactor mass flux at 4500 Ma–about three and four times greater than the Neukum and Werner chronologies, respectively–which rapidly decreases with time to intersect the Neukum chronology at 4420 Ma, and the Werner chronology at about 4400 Ma. The impactor mass fluxes in the Neukum and Werner chronologies decrease log-linearly with time and intersect each other at ca. 4350 Ma. Total delivered mass in the simulations ranged from 0.0013(*M*_*Earth*_) (0.13 wt% ≈ 7.8 × 10^21^ kg) to 0.0057(*M*_*Earth*_) (0.57 wt% ≈ 3.4 × 10^22^ kg). Intermediate delivered masses of 0.3, 0.4, and 0.5 wt% were also tested in our model.

Total delivered mass for late accretion of 0.57 wt% was selected as the primary scenario for detailed biocompatibility modeling. We did this to adopt a conservative, higher-end estimate of late accretion bombardment intensity that could hamper or thwart prebiotic chemistry from reaching biochemistry. Further, this value was chosen because it aligns with upper-bound constraints derived from the abundances of highly siderophile elements (HSEs) in Earth’s mantle, which have been used to suggest that late accretion augmented Earth’s present mass by roughly 0.5%^61–63^. On the other hand, dynamical models combined with lunar cratering records point to a cumulative mean late accretion mass closer to 0.13–0.3 wt%^25^; our decision using 0.57 wt% allows for an assessment of the worst-case scenario for thermal effects and sterilization. This conservative approach ensures that predictions of biocompatible volumes, hydrothermal activity, and sterilization events provide upper limits on impact disruptions.

The size-frequency distribution of the impactor population used in this study is that of the main asteroid belt^64^ and tested against the lunar cratering record^65^. Impactor diameters range from ~40 to ~500 km, with ~4300 to ~19,000 impactors per simulation depending on delivered mass. Impactor density is approximated at 3000 kg m^-3^ based on the mean bulk density of S-type asteroids^66^, the most common type in the inner asteroid belt^67^. Impactor velocity distributions from Mojzsis et al^25^. were used and the impact angle of each impactor was set to the statistically most probable angle^68^ of 45°. Impactor velocity distribution ranges from 11.3 km s^-1^ to 45.2 km s^-1^, with a median of 19.8 km s^-1^ and a mean of 20.7 km s^-1^, consistent with a typical impact velocity of ~20 km s^-1^ estimated for the Earth during the period of late accretion^69–71^. The velocity distribution file for our simulations is provided in the **Supplementary Online Materials**.

### Target parameters

Our model runs start with either a pre-existing crust or a global magma ocean, formed by either or both residual heat from planet formation and colossal impacts. Target properties are summarized in Supplementary Online Material Table S1. Mean surface temperature and geothermal gradient were assumed as 20 °C and 70 °C km^-1^, respectively^15^. A mean surface temperature of 20 °C for early Earth between 4.5 and 3.5 Ga is reasonable due to geochemical and geological evidence of liquid water and climate models accounting for factors such as solar luminosity and greenhouse gas concentrations^72^. Abramov et al.^35^ provides additional discussion of the range of possible geothermal gradients on Hadean Earth, including results from the application of the impact bombardment model to a geothermal gradient of 13 °C km^-1^. In this study, we focus on the higher geotherm value of 70 °C km^-1^ to place upper limits on crust volumes melted by impact bombardment.

In calculating post-impact temperature distributions and their subsequent thermal evolution, the thermal and physical processes of basalt were used because it is the dominant rock type in the oceanic crust and is associated with the early stages of crust formation. Arguably, early Earth was likely characterized by abundant mafic crust with some amount of granitic (continental) component as supported by geochemical evidence from ancient zircons^73^ and theoretical studies of early Earth’s tectonic regime^18^. Regardless, the different possible thermal responses of different types of target rock is subordinate to the total effect of impact heating to the crust.

### Hadean zircon age-resetting model

To benchmark our simulations against documented U-Pb age spectra for Hadean zircons, we simulate age-resetting in zircon grains by coupling thermal models with equations for diffusion of Pb^2+/4+^ in zircon^74,75^. Brought to completion, this process resets the radiogenic Pb age of zircon grains. In our model runs we treat zircon grains as isotropic spheres; an adequate approximation for this modeling study because it simplifies what would otherwise be computationally expensive diffusion calculations for millions of model grains. Since the main focus of our study is to understand the broad impact of late accretion on the age evolution of Earth’s crust, this approximation allows for computationally efficient modeling without significantly compromising the accuracy of the results. Our model calculates the diffusion coefficient and the diffusive length scale based on temperature and time step duration. The diffusion coefficient is calculated following Cherniak and Watson^74^ as:

3 $$D=1.1\times {10}^{-1}\,\exp \left(\frac{-550\pm 30\,{kJ}\,{{mol}}^{-1}}{{RT}}\right)\,{m}^{2}{s}^{-1}$$

where *D* is the diffusion coefficient, *R* is the universal gas constant, and *T* is absolute temperature. The distance diffused by Pb in one direction during time *t* can be approximated by:

4 $$X\approx \sqrt{{Dt}}$$

The diffusion coefficient *D* is calculated using Eq. (3), with temperature *T* obtained from the thermal model. This formulation describes the kinetics of diffusant (Pb) loss rather than being constrained to the upper temperature field of zircon stability. The total distance diffused by Pb during the simulation is estimated by summing the diffusion distance *X* within each model element in each model time step being output (*t* = 1 Myr). When the total diffusion distance exceeds a grain radius of 50 μm, the grain is considered reset and a new age is assigned to it. Other zircon sizes were tested by Abramov et al.^35^, indicating that the results are not strongly dependent on grain size. Hence, the results presented here are generally applicable to zircon grains within a common size range of ~20–200 μm.

### Biocompatibility metrics

We extend the thermal outputs of our global 3-D crustal model to compute volumes defined as <110 °C for a loose upper limit on biological activity. This temperature is substantially higher than the thermal stability thresholds of nearly all biomolecules (Table S2), and we chose it as a first pass of our model to analyse the magnitude and range of crustal habitats that would exist for hyperthermophilic microbial communities^76^. Our model also mapped regions that we defined as hydrothermal zones (>140°C/km gradients), so-called “never-sterilized fractions”, and cluster counts of these different categories scaled to 5% land exposure in underground biocompatible niches within 300 m of the surface. As a sensitivity test, we also raised the upper temperature limit to 130°C and found that this does not change the outcome of our analysis. Hydrothermal zones are identified as regions with temperatures <110 °C but with steep vertical thermal gradients (>140 °C/km), indicative of impact-induced hydrothermal circulation that could drive fluid flow, mineral precipitation, and energy gradients favorable for prebiotic synthesis and disequilibrium chemistry for nascent metabolism^50^. We tagged domains as “never-sterilized fractions” to track cumulative maximum temperatures in each model volume element for persistent underground niches that never exceed 110 °C after a specified time. Temperatures for biomolecule stability are based on thermal degradation thresholds reported in Table S2: DNA at <140 °C^77,78^, and RNA-DNA complex stability at <60 °C^79^. What we term “cluster counts” in the model are defined as quantified non-interconnected hydrothermal zones identified and evaluated using the Density-Based Spatial Clustering of Applications with Noise (DBSCAN) algorithm. The DBSCAN algorithm was selected for its ability to discover clusters of arbitrary shape in noisy spatial data^80^ by grouping nearby elements that meet the hydrothermal criteria parameterized in our model into “clusters” which in turn provide a proxy for isolated biocompatible niches within the crust.

## Supplementary information

Supplementary Information

Peer Review File

## Data Availability

All data are available in the main text or the supplementary materials.
