# Supplementary material for: A Hadean timeline for the emergence of the RNA World
Source: Nat Commun. 2026 Sep 22;17:9790. doi: 10.1038/s41467-026-76978-3 (PMC13597498; doi:10.1038/s41467-026-76978-3)
Supplement: Supplementary file 1 [file 41467_2026_76978_MOESM1_ESM.pdf]

Table S1. Summary of target properties used in our simulations. A basaltic target lithology for the Hadean crust is assumed. Thermal conductivity is given in the HEATING materials library[1]; references and/or justifications for other values are provided in the text.

Parameter	Value(s)	Units
Pressure derivative of the bulk modulus	5.5	Unitless
Adiabatic bulk modulus at zero pressure	19.3×10^{9}	Pa
Heat capacity	800	J kg^{-1} °C^{-1}
Density	3000	kg m^{-3}
Latent heat of fusion	400	kJ kg^{-1}
Liquidus temperature	1250	°C
Solidus temperature	1100	°C
Surface temperature	20	°C
Geothermal gradient	70	°C km^{-1}
Thermal conductivity	2.5	W m^{-1} °C^{-1}

Table S2. Summary of activity and synthesis temperatures for relevant biomolecules.

Biomolecule name	Highest temperature of activity (Celsius)	Lowest temperature of activity (Celsius)	Temperature for synthesis
RNA	When exposed to moisture: few weeks activity at room temperature[2]. Half life of 10 min at 100 °C (tRNA). 1 min for mRNA.	Can be stored at extremely low temperatures (-80 °C). Only storage, not active use[2].	Enzymatic depends on the enzyme, typically 20-40 °C. Abiotic 40-80 °C (short stability, chains start to degrade)[3]
DNA	Under dry conditions, completely degrades[4] at 190 °C	Can be stored at extremely low temperatures (-80 °C). Only storage, not active use	Enzymatic: depends on the enzyme (in vivo - 31-37 °C, PCR 70-80 °C)
RNA-DNA duplex	Melting temperature varies depending on the nucleotides and length, but typically[5] between 30-60 °C		
Nucleobases			Uracil in laboratory: 50-110 °C, at different steps of the synthesis. Thymine synthesis 100-140 °C (Choughuley et al.[6])
ATP	80-100-120 °C - ATP half-life few minutes, Arrhenian behaviour[7]	Stored at -20 or -80 °C in aqueous solutions (not active, but does not harm activity in the long run)[8]	Phosphorylation of ADP with Fe^{3+} - 20-30 °C. Acetyl-phosphate (precursor) synthesis 20 °C at pH 7, 50 °C at pH 8 (Kitani et al.[9])
Proteins	There are thermostable proteins (active at 80 °C, degrades at 101 °C) (Kulkarni et al.[10]). Limit of life generally defined[11] as 110-200 °C	Lowest temperature with life -50 °C.	In vivo: Growth rate optimum, 37 °C for mesophiles (like E. coli),
Amino-acids	Most amino-acids are stable till 120 °C (Weder and Sohns[12])	-	Around 220 °C (Zhang et al.[13])
Fatty acid bilayers	High tolerance for high temperatures (80-90 °C) with ether bonds (typical in archaea)[14]	They can stay fluid even at low temperatures (-60 °C) in case of unsaturated lipids	Room temperature is ideal for bilayer formation[15]

Table S3. Time series of potentially ocean-sterilizing impacts (400-500 km impactor diameter range). Total delivered mass is 0.57 wt% of Earth.

Bin (Ga)	Number of impacts in simulation
4.5-4.49	6
4.49-4.48	7
4.48-4.47	5
4.47-4.46	3
4.46-4.45	4
4.45-4.44	4
4.44-4.43	3
4.43-4.42	1
4.42-4.41	2
4.41-4.40	1
4.40-4.39	1
4.39-4.38	0
4.38-4.37	1
4.37-4.36	0
4.36-4.35	0
4.35-4.34	0
4.34-4.33	0
4.33-4.32	1
4.32-4.31	0
4.31-4.30	0
4.30-4.29	1

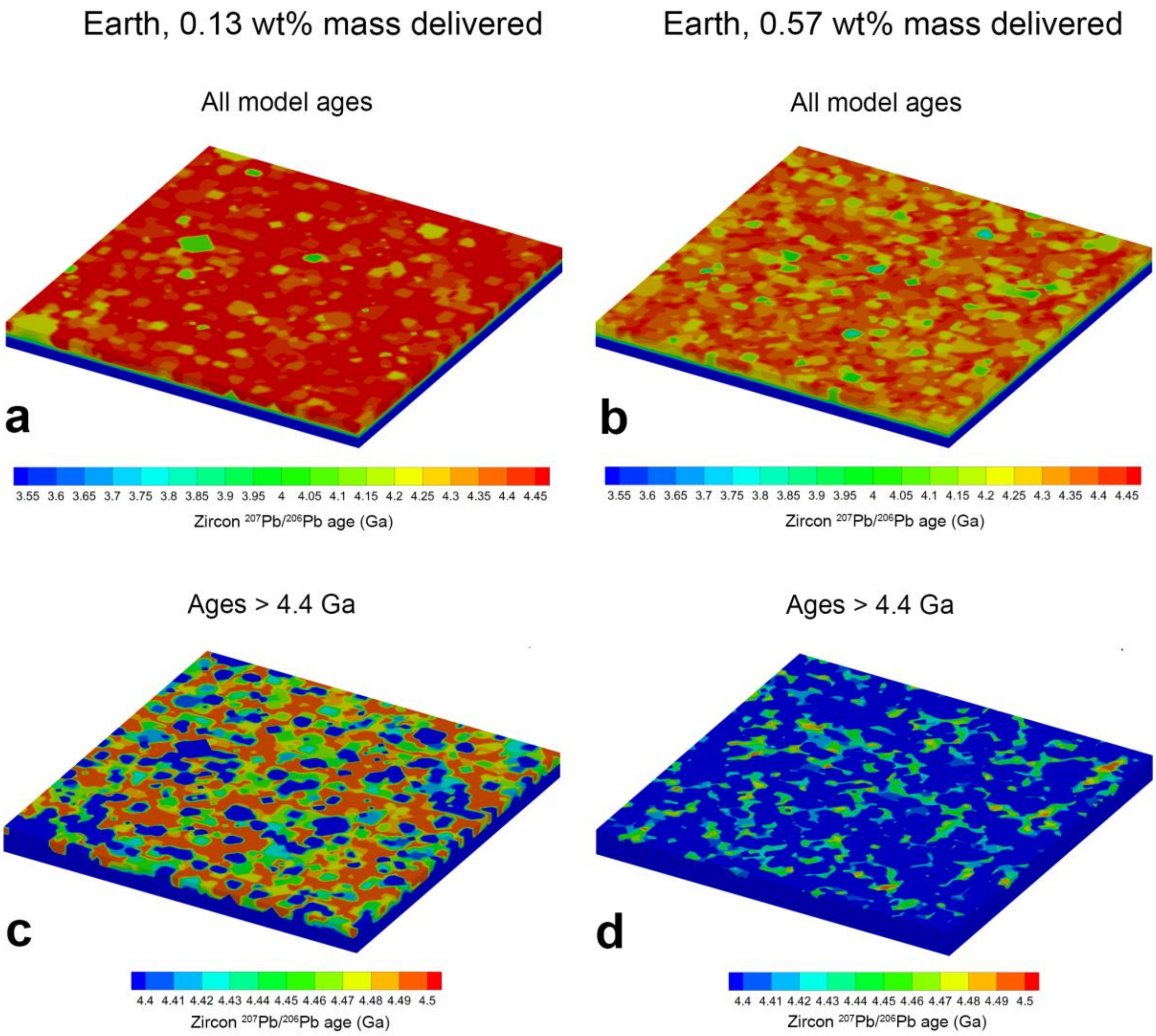

Figure S1. Predicted crustal age distributions derived from model runs from 4.5 Ga to 3.5 Ga. Only the upper 25 km of the crust is shown. (a) Zircon ages for 0.13 wt% delivered mass. (b) Zircon ages for 0.57 wt% delivered mass. (c) Zircon ages between 4.4 to 4.5 Ga for 0.13 wt% delivered mass, indicating predicted age distribution of the oldest crust. (d) Zircon ages between 4.4 to 4.5 Ga for 0.57 wt% delivered mass.

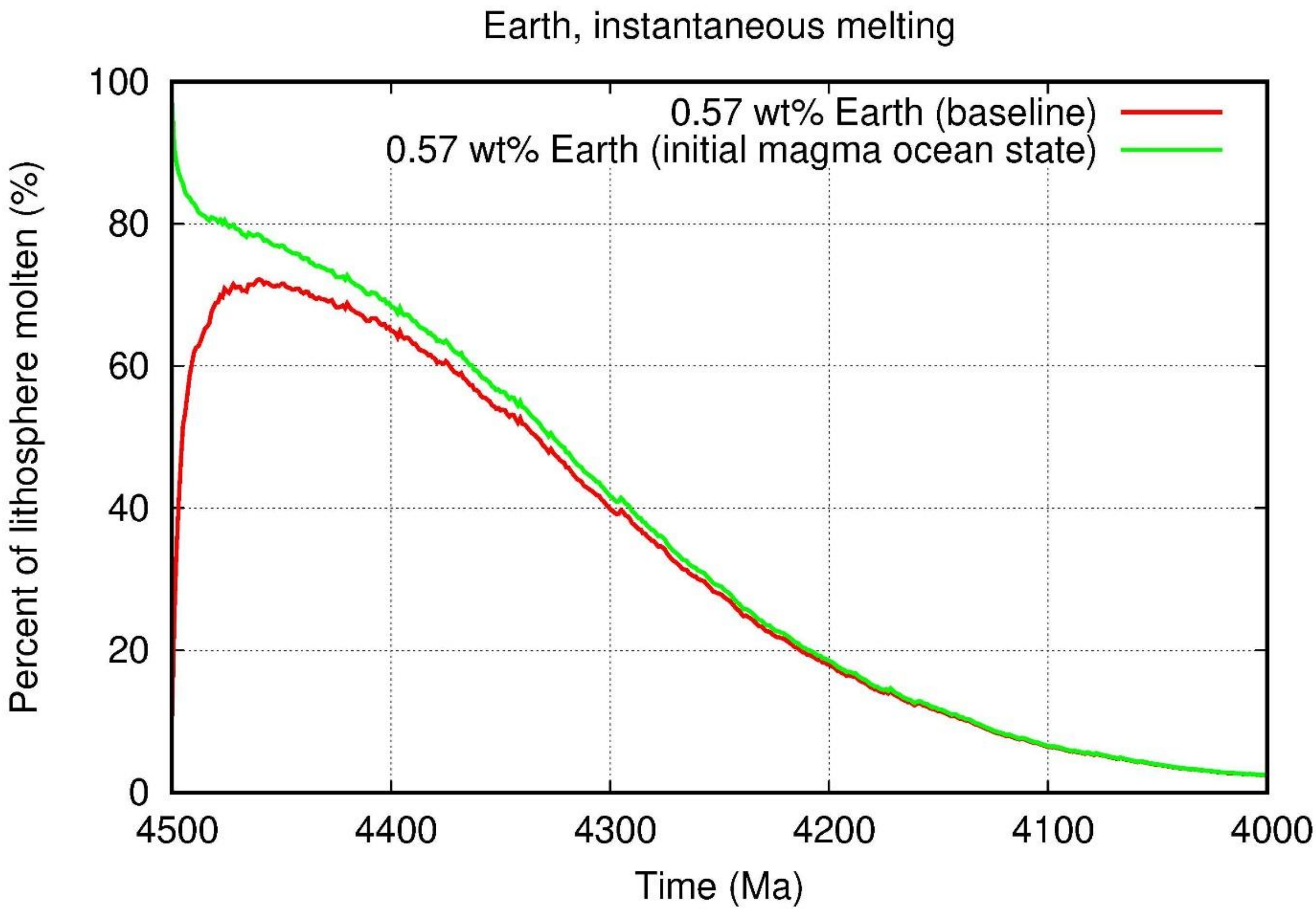

Figure S2. Percent of lithosphere molten at any given time for 0.57 wt% delivered mass, comparing the baseline (pre-formed crust) vs initial magma ocean scenarios. The data used to construct this figure are reported in **Table S3**.

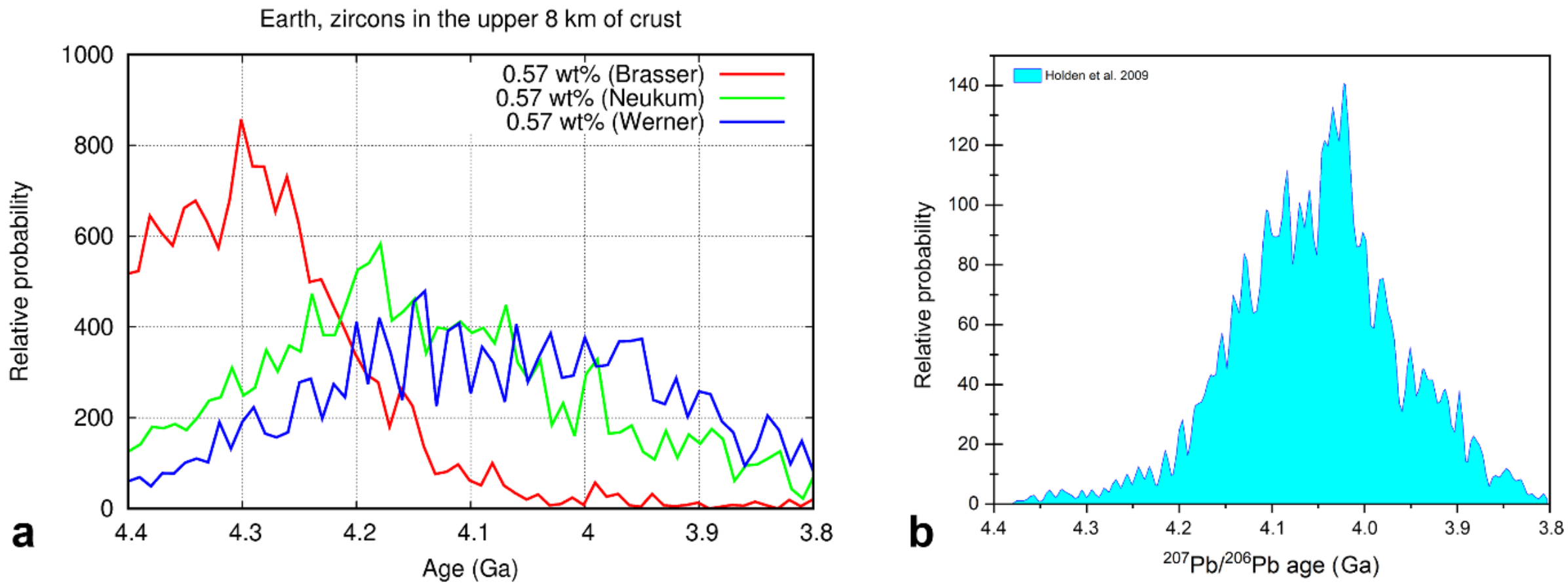

Figure S3. (a) Distribution of zircon ages in the upper 8 km of the crust for Brasser, Neukum, and Werner chronologies at 0.57 wt% delivered mass. (b) Age distribution of ancient terrestrial zircons from Holden et al.[16]

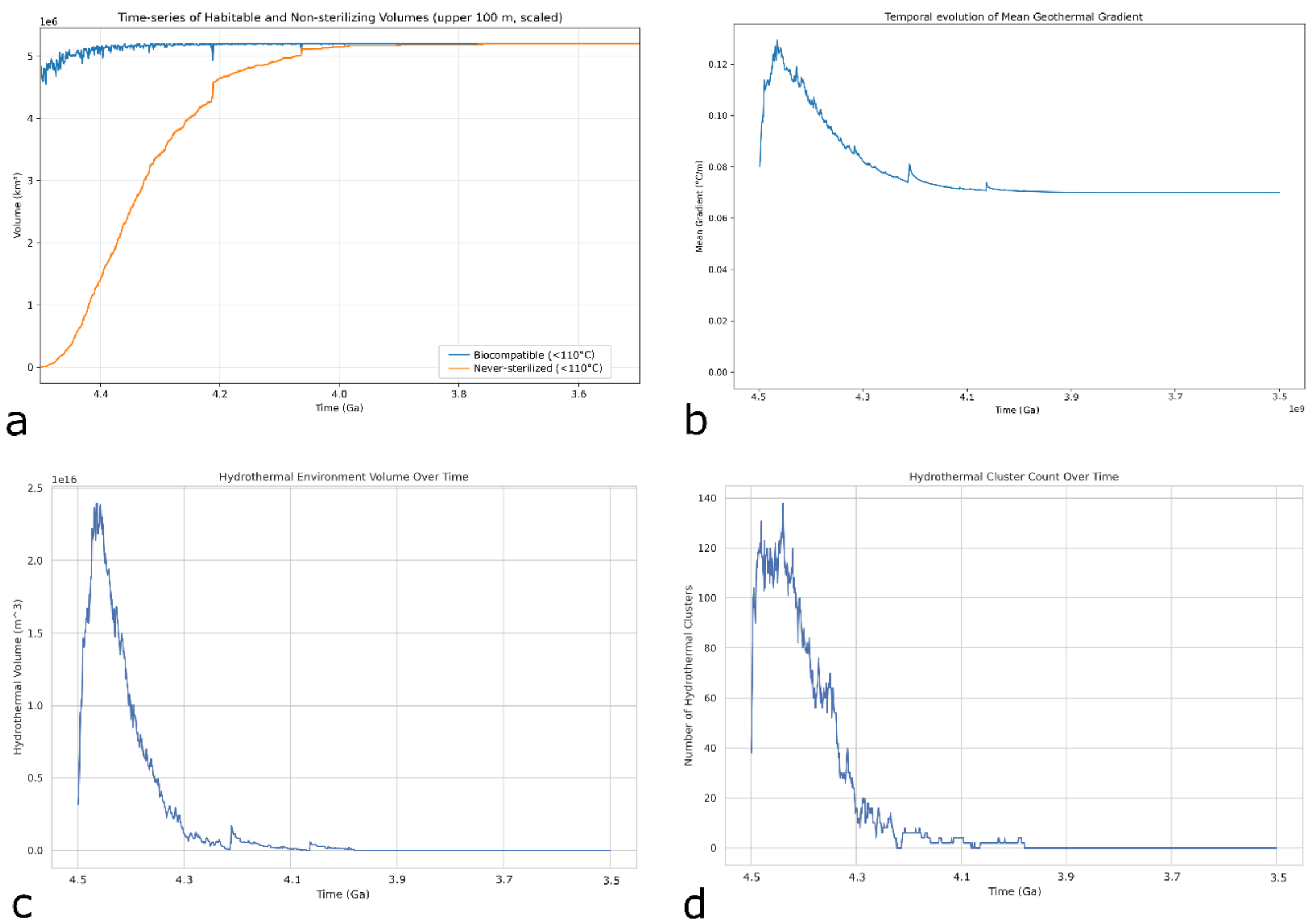

Figure S4. Key results for the 0.13 wt% delivered mass (low end of the range tested), pre-formed crust scenario for comparison. (a) Time series of never-sterilized volume (volume that never exceeds 110 °C after a time specified on the x-axis) in the upper 100 m of the Hadean crust. Biocompatible volume (volume at temperatures below 110 °C) is shown for comparison. (b) Temporal evolution of the mean geothermal gradient of the Hadean and Eoarchean crust over 1 Gyr of simulation time in the upper 4 km of the crust. (c) Hydrothermal volume (< 110 °C and > 140 °C/km thermal gradients) in the upper 100 m of the Hadean crust. (d) Number of hydrothermal clusters (non-interconnected hydrothermal zones) with time.

References

1. Childs, K. W. Heating 7.2 user's manual. ORNL/TM-12262 (1993).
2. Fabre, A.-L., Colotte, M., Luis, A., Tuffet, S. & Bonnet, J. An efficient method for long-term room temperature storage of RNA. *Eur. J. Hum. Genet.* **22**, 379–385 (2014).
3. Hassenkam, T. & Deamer, D. Visualizing RNA polymers produced by hot wet-dry cycling. *Sci. Rep.* **12**, 10098 (2022).
4. Karni, M., Zidon, D., Polak, P., Zalevsky, Z. & Shefi, O. Thermal degradation of DNA. *DNA Cell Biol.* **32**, 298–301 (2013).
5. Kornienko, I. V., Aramova, O. Y., Tishchenko, A. A., Rudoy, D. V. & Chikindas, M. L. RNA stability: a review of the role of structural features and environmental conditions. *Molecules* **29**, 5978 (2024).
6. Choughuley, A. S. U., Subbaraman, A. S. & Kazi, Z. A. A possible prebiotic synthesis of thymine: uracil-formaldehyde-formic acid reaction. *Biosystems* **9**, 73–80 (1977).
7. Moeller, C., Schmidt, C., Guyot, F. & Wilke, M. Hydrolysis rate constants of ATP determined in situ at elevated temperatures. *Biophys. Chem.* **290**, 106878 (2022).
8. Chen, Y. & Xia, S. Determination of the stability of plasma ATP in vitro. *Am. J. Blood Res.* **11**, 96–99 (2021).
9. Kitani, A., Tsunetsugu, S. & Sasaki, K. FeIII-ion-catalysed non-enzymatic transformation of ADP into ATP. *J. Chem. Soc., Perkin Trans. 2*, 329–331 (1991).
10. Kulkarni, T. S. et al. Crystal structure of β-glucosidase 1A from Thermotoga neapolitana and comparison of active site mutants for hydrolysis of flavonoid glucosides. *Proteins* **85**, 872–884 (2017).
11. Brock, T. D. Life at high temperatures. *Science* **230**, 132–138 (1985).

12. Weder, J. K. & Sohns, S. Model studies on the heating of food proteins. Amino acid composition of lysozyme, ribonuclease and insulin after dry heating. *Z. Lebensm. Unters. Forsch.* **176**, 421–425 (1983).

13. Zhang, Z. et al. Evaluating the abiotic synthesis potential and the stability of building blocks of life beneath an impact-induced steam atmosphere. *Front. Microbiol.* **14**, 1032073 (2023).

14. Saracco, M. et al. Bilayer-forming lipids enhance archaeal monolayer membrane stability. *Int. J. Mol. Sci.* **26**, 3045 (2025).

15. Shields, A., Abbey, W. & Vance, S. The effect of temperature on fatty acid vesicle formation in simulated deep-ocean conditions. *AGU Fall Meeting Abstracts* **2011**, P21B-1662 (2011).

16. Holden, P. et al. Mass-spectrometric mining of Hadean zircons by automated SHRIMP multi-collector and single-collector U/Pb zircon age dating: the first 100,000 grains. *Int. J. Mass Spectrom.* **286**, 53–63 (2009).
