# Supplementary material for: A Hadean timeline for the emergence of the RNA World
Source: Nat Commun. 2026 Sep 22;17:9790. doi: 10.1038/s41467-026-76978-3 (PMC13597498; doi:10.1038/s41467-026-76978-3)
Supplement: Supplementary file 2 [file 41467_2026_76978_MOESM2_ESM.pdf]

nature portfolio

Peer Review File

A Hadean timeline for the emergence of the RNA World

Corresponding Author: Professor Stephen Mojzsis

This file contains all reviewer reports in order by version, followed by all author rebuttals in order by version.

Version 0:

Reviewer comments:

Reviewer #1

(Remarks to the Author)
This is a very interesting and potentially important manuscript. The central premise, exploring the thermal stability of biomolecules under plausible geologic conditions, is compelling. As far as I am aware, the way the authors have constructed this problem, particularly with explicit emphasis on geologic processes and early Earth environments, is novel. This is one of those papers where the reader may reasonably wonder why this question has not been addressed in this way before. My comments below are intended to help the authors clarify the framing, broaden the interpretive reach of the manuscript, and make several modeling choices more transparent for a broad Nature Communications readership. I recommend publication after minor revisions, largely at the authors' discretion
-The manuscript suggests that an RNA World emerged and that more than 150 million years then passed before the emergence of LUCA. This is an interesting inference, but the interval feels extremely long in the context of origin-of-life discussions. The authors should consider explaining the rationale for this timescale more explicitly, especially where this point is introduced near the end of the abstract. Is this interval required by the model, inferred from the impact chronology, or adopted from prior constraints on LUCA? Clarifying this would strengthen the broader implications of the paper.
-The authors lean strongly into the RNA World hypothesis as the relevant prerequisite for evaluating whether the early Earth was biocompatible. This is reasonable, but it may unnecessarily narrow the applicability of the study. The paper could be strengthened by briefly discussing whether the results also bear on metabolism-first origin-of-life models (or others). For example, are the molecular components or intermediates associated with primitive carbon-fixation cycles also expected to be thermally stable or unstable under the modeled conditions? If the authors regard metabolism-first models as outside the scope of the manuscript, or as unreasonable in this context, that should be stated more clearly. Otherwise, a short discussion could broaden the significance of the work beyond RNA World scenarios alone.
-The authors state that the Hadean Earth was generally ill-suited for maintaining appropriate conditions for life. I think this statement would be more effective if the authors clearly define what they mean by "ill-suited." Several studies have argued that persistent volumes of liquid water may have existed deep into the Hadean. In addition, other work, including work by some of the present authors, has suggested that Earth's early bombardment history may have been relatively temperate compared with older views. The authors should clarify whether they mean globally inhospitable, intermittently inhospitable, locally inhospitable near impact sites, or specifically inhospitable for preserving thermally fragile RNA World molecules.
-The statement that the Last Universal Common Ancestor of the contemporary DNA world was already in place by ca. 4.2 Ga is important for the manuscript's overall argument, but 4.2 Ga is a very specific age to assign to LUCA. The authors should provide more context for how robust this number is. Is this age intended as a hard constraint, a plausible upper bound, or one estimate among several? I understand why this value supports the paper's broader conclusions, but the uniqueness and precision of the age are not immediately clear.
-The authors compare their predicted impact-induced zircon geochronology to published age spectra of Hadean zircons from the Jack Hills. It would be useful to explain why the comparison is limited to Jack Hills rather than the broader Hadean zircon population. In addition, several studies have shown that the age distribution of ancient grains can vary substantially depending on which Jack Hills unit is sampled. The authors should clarify how this sampling heterogeneity affects the comparison.
-When the authors refer to zircon age resetting by Pb loss, it would be useful to specify exactly what quantity or model output is being used. E.g., what are D*t values used, and is T constrained at the upper field of zircon stability? A clearer description would help readers understand how the modeled thermal histories are being translated into apparent zircon ages.
-The manuscript states: "It should be emphasized that these model ages only apply to impact processes as simulated in our work and do not account for normal magmatic processes that generate zircon-saturated magmas." This is an important clarification, but the meaning could be expanded. Does this mean that the modeled age spectra represent only impact-generated zircon crystallization or resetting, while excluding background crustal magmatism that could independently

generate zircon-saturated melts? If so, the authors should make clear how this limitation affects comparison with natural Hadean zircon populations, which likely integrate multiple zircon-forming and zircon-modifying processes.
-The phrase "masses in the stagnant lid scenario where no intrinsic rock recycling process is operative" seems too strong. Even in a stagnant-lid setting, impact melting, excavation, burial, and redistribution of crustal material represent forms of rock recycling. The authors may wish to revise this phrasing to distinguish between plate-tectonic recycling and impact-driven crustal reworking.

-The manuscript discusses thermal boundary layers and crustal temperatures at depths of around 4 km, but later quantifies hydrothermal or biocompatible zones in the upper 300 m of crust. The connection between these depth regimes should be made more explicit. In particular, the authors should explain how they map from the modeled impact thermal behavior at greater depth to the shallow geothermal gradients and temperature fields shown in Figures 5, 6, and 7. This issue is especially relevant for the statement: "Turning now to the habitats that could serve as the crucible for an RNA World, Figure 5 quantifies hydrothermal zones, i.e., high geothermal gradients >140°C km^{-1}, temperature fields <110°C, within the upper 300 m of the crust." The relevance of these shallow zones would be clearer if the authors explicitly connected them to the modeled thermal boundary layers and deeper crustal temperature structure.
-Related to the previous point, the discussion of shallow subsurface habitability would benefit from additional explanation of how the authors mapped the deeper thermal model results onto the shallow surface thermal gradients shown in Figure 7. The same concern applies to the very shallow temperature slices shown in Figure 6. A short methodological clarification may be sufficient.
-The chronologies provided in Supplementary Equations 1 and 2 should be explained in more detail. In particular, it would be useful to know the origin and meaning of parameters such as τ, β, and F_0. These may be standard parameters in the impact-modeling community, but for the broad readership of Nature Communications, the manuscript would be stronger if these values were introduced more explicitly, including their sources and physical significance, and broadly why different choices were made by each of the three different model papers cited.
-I recognize that the authors use a mass accumulation of 0.57% as a worst-case scenario. It would be useful to know how the conclusions change under a more modest mass accumulation scenario. Would this increase the temporal separation between the hypothesized RNA World and LUCA? If so, that would be worth stating explicitly, since it may affect how readers interpret the timing and severity of thermal challenges to early biochemistry.
-In Figure 2, the dark mustard/golden-brown color does not appear to map clearly to the temperature scale. For example, in Figure 2D this color dominates the panel, but it is not clear what temperature range it corresponds to. The figure legend or color bar should be revised so that this color can be interpreted unambiguously.
-Figure 4 shows biocompatible volume in the upper 300 m of the Hadean crust. As noted above, I would like to see additional explanation of how this volume was calculated from the modeled impact thermal behavior, particularly where the main thermal discussion involves depths closer to 4 km. The same clarification would help with the shallow temperature slices shown in Figure 6.
Overall, this is a creative manuscript. The paper asks an important question in a way that feels both geologically grounded and broadly relevant to origin-of-life research. My comments are mostly requests for clarification, and improved explanation of modeling assumptions.

Reviewer #2

(Remarks to the Author)
This is a signed review.
1. I am not an expert on modeling. Therefore, my role in this review is to explain to the editors why this paper is important and interesting to people outside of the field, to the broad readership that Nature Communications seeks to reach.

This is very much the case. The first serious effort following this logic, reference 20 in the manuscript under review, [Benner, S. A. et al. When did life likely emerge on Earth in an RNA-first process?. ChemSystemsChem 2, e1900035 (2020)], is indeed "under-constrained". It gave a date at 4.36 ± 0.1 Ga.

A subsequent paper criticized this model [Wogan, N. F., Catling, D. C., & Zahnle, K. J. (2024). Timing and likelihood of the origin of life derived from post-impact highly reducing atmospheres. Astrobiology, 24(9), 881-891.], but came to the same number, but disagreed on its uncertainty (arguing that it is from 3.9 to 4.45 Ga), but agreed that the larger issue was getting a reducing hydrogen-rich atmosphere, and decided that for a 4 x 10^^20 kg and a 5 x 10^^21 kg impactor, as extremes, the most likely timing is 4.35 Ga.

The manuscript under review is a major advance over both papers, but in particular over Wogan, since it rules out dates before 4.4 Ga.

2. As edits,
2.1 the sentence on page 5 is dangling ("allowed for the first life forms to emerge only on."). Perhaps it is intended to be "on dry land"
2.2. On page 3, line 18, the standard RNA first model does not exclude peptides. It does, however, exclude "encoded peptides". However, the reference cited [Benner, S. A. Detecting Darwinism from molecules in the Enceladus plumes, Jupiter's moons, and other planetary water lagoons. Astrobiology 17, 840–851 (2017).] is probably not the best.
2.3. Wogan et al. should be cited.
2.4. For reference 3, better might be these references
Benner, S.A., Ellington, A. D., Tauer, A. (1989) Modern metabolism as a palimpsest of the RNA world. Proc. Nat. Acad. Sci. 86, 7054-7058.

Benner, S.A., Carrigan, M.A., Ricardo, A., Frye, F. (2006) Setting the stage. The history, chemistry and geobiology behind RNA. in RNA World, Gesteland, R. F., Cech, T. R., Atkins, J. F. editors 1-21
Neveu, M., Kim, H.-J., Benner, S.A. (2013) The "Strong" RNA World hypothesis. Fifty years old. Astrobiology 13, 391-403.

3. Turning to science, some comments relevant to th three issues.
3.1. The central chemical problem for the RNA first model for the origin of life is the discordance between the redox neutral atmosphere that dominated the Hadean, versus the need for reduced nitrogen (at the oxidation state of ammonia) in the nucleobases. The first does not have sufficient ammonia. This is the fundamental flaw with the Sutherland model in reference `. The compounds that Sutherland invokes in the cyanosulfidic hypothesis are simply not available in any standard model for the Earth. Further, Sutherland's chemistry solves no problem it needs solution. The key step is way of getting formaldehyde, but formaldehyde is abundant in the redox neutral atmosphere without his chemistry. The second key step is joining to formaldehyde units to get C2H4O4, but again, the ppm of this compound that is easily formed in the standard redox neutral atmosphere is adequate to get an autocatalytic cycle to make ribose.

Now, impacts are important parts of actionable models to get RNA, other than Sutherland's, but because they generate reducing atmospheres. The bases in RNA become available only if the atmosphere is reduced by the shattered iron core of an impactor, and that place is a lower limit on the size of the useful impactor it also constraints the latest date when life could have emerged. This is noted on page 5 of the manuscript under review, as well as in reference 21. However, except for people who are very familiar with this field, it is likely to get lost.

3.2. Hydrothermal vents are not necessary for any of the actionable models for RNA synthesis. They may be necessary to support a primordial biome, as mentioned on page 6. In fact, all that is necessary for a standard redox neutral atmosphere is a constrained subaerial, intermittently hydrated aquifer. Thus, the prebiotic chemistry line mixes up these two classes of models.

3.3. Notwithstanding, the major reason why this paper is important, is because it constrains the front end of the process, providing an important analysis that indicates where the window opens, after the probability of a sterilizing impactor has ceased. The discussion of Zircon is important and new.

3.4. The molecular clock analysis is fine, although, in Hirakawa (ref. 24) the sequential events relating to origins occurring 100 million years before the clock suggests the divergence of the three kingdoms which occurs 100 million years before the first isotopic. The light carbon reported in Zircon is stated to be "too good to be true". Of course, it may be true.

Reviewer #3

(Remarks to the Author)
Review for Manuskript: "A Hadean Timeline for the Emergence of the RNA World" by Abraham et al. and S. J. Mojzsis. The authors develop in this manuscript a new model describing the point in time at which early life (RNA-based) could have existed on Earth. In particular, the authors address the question of when an RNA world could have stably emerged.
The prerequisites for this are, first, that the Earth's surface or other habitats such as oceans—at not too great depths—had cooled sufficiently to allow RNA molecules to remain stable. Second, the probability of sterilization of the surface by large meteorite impacts had to be low. Only under these conditions can complex prebiotic chemistry proceed in a sufficiently stable manner to allow the formation and accumulation of complex molecules.
Using methods that, as a non-geoscientist, I am unfortunately not in a position to evaluate, the authors conclude that the conditions for an RNA world on Earth may already have been optimal around 4.3 billion years ago. The authors refer to this as a "sweet spot" for the RNA world. This result is highly interesting and exciting, as it places the possible emergence of an RNA world significantly earlier than previously assumed. Based on these new estimates, this implies that RNA molecules may have arisen more rapidly than previously suggested and that complex cellular life consequently had more time to evolve.
Regarding the authors' concept of the RNA world, it is noteworthy that the manuscript continues to treat the molecules required for the origin of life as belonging to two separate domains: RNA and peptides. In 2022, it was proposed that a purely RNA-based world may never have existed, but rather that an RNA–peptide world may have been present from the outset, composed of RNA building blocks that simultaneously carried amino acid functionalities or amino acids themselves. The basis of such a system would be RNA–peptide chimeras. While this distinction is not directly relevant for the publication of the model, it would nevertheless be appropriate to briefly address it in the manuscript.

Thomas Carell

Reviewer #4

(Remarks to the Author)
The manuscript discusses the consequences of terrestrial early bombardments on the origin of life. The writing is clear and well-articulated. However, the study's main conclusions rest on modeling that is quantitatively very similar to work previously published by the same authors (Abramov et al., 2009, 2013). Although the authors have implemented some changes—including the use of different bombardment histories—the overall approach appears to neglect major areas for improvement.

Here are listed some general points to consider:

1. The manuscript emphasizes the predicted timing of habitability (~4.3 Ga), which is reasonable based on prior work. However, the modeling presented here relies on scaling laws similar to those used in earlier studies, representing little significant advancement. For instance, the manuscript does not model hydrothermal alteration, which can significantly outlast predictions based on simplistic temperature scaling laws (see the recent Chicxulub debates; Pickersgill et al., 2026). This discrepancy also applies to the relationship between crustal volumes above given temperatures. Moreover, impact melt scaling laws can be improved by following the methodology outlined by Maske et al., 2025.

2. One critical aspect is the overall delivered mass during late accretion. The manuscript considers masses up to 0.57 wt\% Earth's masses. However, SPH impact simulations have shown that impactor metal retention is not 100% efficient (which is the primary constraint used to derive a late accretion mass), and so a larger mass is likely (Marchi et al., 2018; Citron & Stewart 2022; Itcoviz et al., 2024).

3. The relevance of the Hadean zircon age reset model to the present paper is unclear. Because the final zircon age distribution is not used to constrain the impact flux, it has no bearing on the biocompatible volume.

Open Access This Peer Review File is licensed under a Creative Commons Attribution 4.0 International License, which permits use, sharing, adaptation, distribution and reproduction in any medium or format, as long as you give appropriate credit to the original author(s) and the source, provide a link to the Creative Commons license, and indicate if changes were made.
In cases where reviewers are anonymous, credit should be given to 'Anonymous Referee' and the source.
The images or other third party material in this Peer Review File are included in the article's Creative Commons license, unless indicated otherwise in a credit line to the material. If material is not included in the article's Creative Commons license and your intended use is not permitted by statutory regulation or exceeds the permitted use, you will need to obtain permission directly from the copyright holder.
To view a copy of this license, visit https://creativecommons.org/licenses/by/4.0/

Response to Reviewers' Comments

We sincerely thank all four reviewers for their positive overall assessment, insightful comments, and constructive suggestions. Their collective feedback has helped us clarify key points, improve the framing and transparency of our modelling choices, and broaden the discussion of implications. We have revised the manuscript accordingly and provide point-by-point responses below. A version of the revised manuscript with tracked changes is provided for ease of review.

In the responses below, reviewer comments are *italicized*.

Response to Reviewer #1

We thank Reviewer #1 for the encouraging assessment that this is "a very interesting and potentially important manuscript" with a novel approach, and for the detailed, thoughtful comments aimed at improving clarity and broadening reach. We address each point below.

The manuscript suggests that an RNA World emerged and that more than 150 million years then passed before the emergence of LUCA. This is an interesting inference, but the interval feels extremely long in the context of origin-of-life discussions. The authors should consider explaining the rationale for this timescale more explicitly, especially where this point is introduced near the end of the abstract. Is this interval required by the model, inferred from the impact chronology, or adopted from prior constraints on LUCA? Clarifying this would strengthen the broader implications of the paper.

We thank the reviewer for this helpful observation. The Moody et al. (2024) LUCA age range is reported as 4.09-4.33 Ga in that study, and this full window is already cited in other sections of the manuscript. We agree that the time between the RNA World and the emergence of LUCA is highly uncertain, as it depends on numerous evolutionary steps that are difficult to constrain from either the geological or phylogenetic record. Combining our model-derived sweet spot timing of ~4.33 Ga with the LUCA window of 4.09-4.33 Ga suggests a possible interval of ~0 to ~240 Myr. For comparison, the earliest tentative evidence of life dates to 4.10 ± 0.01 Ga (Bell et al., 2015), which we cite in our manuscript. To address the reviewer's concern about this interval and to avoid introducing any unsubstantiated point estimate, we have edited the manuscript to (1) replace the "ca. 4.2 Ga" references with the complete 4.09-4.33 Ga window from Moody et al. (2024), or indicate that "4.2 Ga" is the approximate mean of this window where appropriate, and (2) replace "~150 Myr" with "~130 Myr", and explicitly state that it represents an average value.

The authors lean strongly into the RNA World hypothesis as the relevant prerequisite for evaluating whether the early Earth was biocompatible. This is reasonable, but it may unnecessarily narrow the applicability of the study. The paper could be strengthened by briefly discussing whether the results also bear on metabolism-first origin-of-life models (or others). For example, are the molecular components or intermediates associated with primitive carbon-fixation cycles also expected to be thermally stable or unstable under the modeled conditions? If the authors regard metabolism-first models as outside the scope of the

manuscript, or as unreasonable in this context, that should be stated more clearly. Otherwise, a short discussion could broaden the significance of the work beyond RNA World scenarios alone.

We appreciate the suggestion to broaden the scope, and we agree that the metabolism-first hypothesis deserves explicit mention. The core distinction is that metabolism-first models posit self-sustaining, energy-driven chemical reaction networks (rather than photosynthesis, which relies on energy from the Sun, these were powered by geothermal energy, mineral catalysts, and proton gradients) as the starting point, with genetic information-storage molecules arising later. These energy sources are directly analogous to the impact-generated hydrothermal oases that our model shows became persistent after ~4.4 Ga. While the thermal stability thresholds for organic compounds themselves may not differ dramatically between the two frameworks, the efficiency and specific pathways of the underlying reaction networks are expected to be strongly temperature-dependent. We have added a short discussion to acknowledge this point and to note that the persistent mild-temperature, disequilibrium chemistry-rich niches identified in our simulations after 4.4 Ga would have provided suitable conditions for either class of model (or for hybrid RNA-peptide scenarios), although the magnitude of temperature effects on prebiotic reaction kinetics is difficult to constrain with confidence given our current understanding.

The authors state that the Hadean Earth was generally ill-suited for maintaining appropriate conditions for life. I think this statement would be more effective if the authors clearly define what they mean by "ill-suited." Several studies have argued that persistent volumes of liquid water may have existed deep into the Hadean. In addition, other work, including work by some of the present authors, has suggested that Earth's early bombardment history may have been relatively temperate compared with older views. The authors should clarify whether they mean globally inhospitable, intermittently inhospitable, locally inhospitable near impact sites, or specifically inhospitable for preserving thermally fragile RNA World molecules.

We have revised the Introduction to clarify, replacing "ill-suited" with "globally inhospitable", as suggested by the reviewer. Additionally, we have replaced "the Hadean Earth was generally" with "the Earth was initially" to clarify that we are not referring to the entire Hadean Eon.

The statement that the Last Universal Common Ancestor of the contemporary DNA world was already in place by ca. 4.2 Ga is important for the manuscript's overall argument, but 4.2 Ga is a very specific age to assign to LUCA. The authors should provide more context for how robust this number is. Is this age intended as a hard constraint, a plausible upper bound, or one estimate among several? I understand why this value supports the paper's broader conclusions, but the uniqueness and precision of the age are not immediately clear.

We have added additional context by specifying that the 4.2 Ga figure is the approximate mean of the 4.09-4.33 Ga range taken from the recent molecular-clock study of Moody et al. (2024). Additionally, we have replaced "4.2 Ga" with the full range (4.09-4.33 Ga) in other locations in the manuscript to clarify.

The authors compare their predicted impact-induced zircon geochronology to published age spectra of Hadean zircons from the Jack Hills. It would be useful to explain why the comparison is limited to Jack Hills rather than the broader Hadean zircon population. In addition, several studies have shown that the age distribution of ancient grains can vary

substantially depending on which Jack Hills unit is sampled. The authors should clarify how this sampling heterogeneity affects the comparison.

We have expanded discussion of Jack Hills zircons to explain that this dataset is the largest, oldest and most extensively studied Hadean zircon population, making it the natural benchmark. We have additionally revised the paper to acknowledge that zircon age distributions can vary between different Jack Hills units: some sections are dominated by Archean grains, while others contain Proterozoic zircons (as young as ~1.58 Ga) and lack or have very few >4.0 Ga grains (e.g., Cavosie et al. 2004). We clarify that we focus on the oldest (Hadean) zircon population, in which the same ~4.0-4.1 Ga peak seen in Holden et al. (2009) has been independently measured (e.g., Cavosie et al. 2004; Wang & Wilde, 2018), making our model-data comparison robust at the level of overall shape. There are also data for the "Green Sandstone Bed" in the Barberton Greenstone Belt which report Hadean zircon ages. Yet, these data add little to the story and indeed skew towards ages peaks *younger* than that of the Jack Hills' population (cf. Drabon and co-authors).

*When the authors refer to zircon age resetting by Pb loss, it would be useful to specify exactly what quantity or model output is being used. E.g., what are D*t values used, and is T constrained at the upper field of zircon stability? A clearer description would help readers understand how the modeled thermal histories are being translated into apparent zircon ages.*

We have revised the relevant subsection in the Methods ("Hadean zircon age-resetting model") to clarify the description of the zircon age-resetting model and (i) explain how diffusion coefficient D is calculated, and (ii) explicitly state the duration of time t as 1 Myr. We have also clarified that this model does not concern the "upper field of zircon stability" but instead the kinetics of diffusant loss and thus variable loss of daughter product.

The manuscript states: "It should be emphasized that these model ages only apply to impact processes as simulated in our work and do not account for normal magmatic processes that generate zircon-saturated magmas." This is an important clarification, but the meaning could be expanded. Does this mean that the modeled age spectra represent only impact-generated zircon crystallization or resetting, while excluding background crustal magmatism that could independently generate zircon-saturated melts? If so, the authors should make clear how this limitation affects comparison with natural Hadean zircon populations, which likely integrate multiple zircon-forming and zircon-modifying processes.

We have expanded the paragraph in question to clearly state that the synthetic age spectra represent only impact-generated crystallization or Pb-loss resetting. They deliberately exclude background magmatic zircon formation from intrinsic processes such as mantle melting or intracrustal melting. Consequently, our model spectra constitute an "impact-only" end-member. The fact that even the high-mass (0.57 wt%) runs produce age peaks *older* than the observed Jack Hills distribution (and indeed, of the Green Sandstone Bed zircons) implies that additional non-impact processes were required to generate the younger zircons that dominate the natural record. We have revised the manuscript to state this more clearly.

The phrase "masses in the stagnant lid scenario where no intrinsic rock recycling process is operative" seems too strong. Even in a stagnant-lid setting, impact melting, excavation, burial, and redistribution of crustal material represent forms of rock recycling. The authors may wish to revise this phrasing to distinguish between plate-tectonic recycling and impact-driven crustal reworking.

We agree the original phrasing was too absolute. We have revised it to: "...masses due to thermal effects of impacts on a stagnant-lid crust with no plate tectonics-like recycling".

The manuscript discusses thermal boundary layers and crustal temperatures at depths of around 4 km, but later quantifies hydrothermal or biocompatible zones in the upper 300 m of crust. The connection between these depth regimes should be made more explicit. In particular, the authors should explain how they map from the modeled impact thermal behavior at greater depth to the shallow geothermal gradients and temperature fields shown in Figures 5, 6, and 7. This issue is especially relevant for the statement: "Turning now to the habitats that could serve as the crucible for an RNA World, Figure 5 quantifies hydrothermal zones, i.e., high geothermal gradients >140°C km^{-1}, temperature fields <110°C, within the upper 300 m of the crust." The relevance of these shallow zones would be clearer if the authors explicitly connected them to the modeled thermal boundary layers and deeper crustal temperature structure.

Related to the previous point, the discussion of shallow subsurface habitability would benefit from additional explanation of how the authors mapped the deeper thermal model results onto the shallow surface thermal gradients shown in Figure 7. The same concern applies to the very shallow temperature slices shown in Figure 6. A short methodological clarification may be sufficient.

Figure 4 shows biocompatible volume in the upper 300 m of the Hadean crust. As noted above, I would like to see additional explanation of how this volume was calculated from the modeled impact thermal behavior, particularly where the main thermal discussion involves depths closer to 4 km. The same clarification would help with the shallow temperature slices shown in Figure 6.

We have added a new methodological clarification paragraph (at the end of "Impact bombardment model" subsection) which addresses the issues raised by the reviewer by describing the spatial and temporal resolution of the model and the approximations involved: "The three-dimensional model is composed of 76 × 76 × 34 nodes, corresponding to horizontal inter-node distance of ~300 km and vertical inter-node distance of ~4 km. Near-surface geothermal gradients and temperatures within 300 m of the surface are calculated by linearly interpolating between the top two nodes of the model. This approximation is valid at the output frequency of the model (1 million years), at which time the upper 4 km temperature-vs-depth curve would be expected to be linear to within ~2%."

The chronologies provided in Supplementary Equations 1 and 2 should be explained in more detail. In particular, it would be useful to know the origin and meaning of parameters such as τ, β, and F_0. These may be standard parameters in the impact-modeling community, but for the broad readership of Nature Communications, the manuscript would be stronger if these values were introduced more explicitly, including their sources and physical significance, and broadly why different choices were made by each of the three different model papers cited.

We have substantially expanded the "Bombardment parameters" subsection of the Methods. We now explain: (i) the physical meaning of τ (characteristic e-folding timescale of impactor flux decay, reflecting dynamical depletion of the leftover planetesimal population); (ii) β = 0.8 in the Brasser stretched-exponential form, which arises from a combination of impacts onto the planets, impacts onto the Sun and ejection from the Solar System; (iii) F_0 as a

normalization constant chosen so that the integrated delivered mass between 4.5 and 3.5 Ga equals the target value (0.13-0.57 wt%); and (iv) why the three chronologies differ in both functional form and characteristic timescale.

I recognize that the authors use a mass accumulation of 0.57% as a worst-case scenario. It would be useful to know how the conclusions change under a more modest mass accumulation scenario. Would this increase the temporal separation between the hypothesized RNA World and LUCA? If so, that would be worth stating explicitly, since it may affect how readers interpret the timing and severity of thermal challenges to early biochemistry.

We added a supplementary figure (Figure S4) showing key diagnostics for the 0.13 wt% case. Under this lower mass flux, the "sweet spot" occurs ~70 Myr earlier, at ~4.4 Ga. We have added a new paragraph to the paper to state this explicitly.

In Figure 2, the dark mustard/golden-brown color does not appear to map clearly to the temperature scale. For example, in Figure 2D this color dominates the panel, but it is not clear what temperature range it corresponds to. The figure legend or color bar should be revised so that this color can be interpreted unambiguously.

We thank the reviewer for drawing attention to this potential source of confusion. The dark mustard/golden-brown color in Figure 2 is not part of the temperature scale. Rather, it is used to mark the locations and approximate sizes of impact craters (as stated in the original caption: "Dark circles indicate crater locations and approximate final diameters"). To eliminate any ambiguity, we have revised the figure caption to add "these symbols are overlaid on the temperature field and do not correspond to temperature values" and "Note the progressive saturation of the surface by impact craters in later panels."

Response to Reviewer #2

We thank Reviewer #2 for the strong endorsement that this work represents "a major advance" over prior timeline estimates (Benner 2020; Wogan et al. 2024) by ruling out pre-4.4 Ga dates, and for the detailed editorial and scientific suggestions. We address each point in turn.

2.1 the sentence on page 5 is dangling ("allowed for the first life forms to emerge only on."). Perhaps it is intended to be "on dry land"

We have corrected this sentence to: "…allowed for the first life forms to emerge early on."

2.2. On page 3, line 18, the standard RNA first model does not exclude peptides. It does, however, exclude "encoded peptides". However, the reference cited [Benner, S. A. Detecting Darwinism from molecules in the Enceladus plumes, Jupiter's moons, and other planetary water lagoons. Astrobiology 17, 840–851 (2017).] is probably not the best.

We have clarified the sentence to read: "…without the need for other RNAs or for encoded peptides." We have also replaced reference 3 with the three papers recommended by the reviewer (Benner et al., 1989; Benner et al., 2006; Neveu et al., 2013) and at the discretion of the editors.

2.3. Wogan et al. should be cited.

We have added a citation to Wogan et al. (2024) in the Introduction where previous timeline estimates are discussed, noting that this work reached a similar central age (~4.35 Ga) but with broader uncertainty and an emphasis on a reducing hydrogen-rich atmosphere. We emphasize that our approach to the problem of when abiogenesis could occur is markedly different from how Wogan et al. (2024) approached the problem.

2.4. For reference 3, better might be these references
Benner, S.A., Ellington, A. D., Tauer, A. (1989) Modern metabolism as a palimpsest of the RNA world. Proc. Nat. Acad. Sci. 86, 7054-7058.
Benner, S.A., Carrigan, M.A., Ricardo, A., Frye, F. (2006) Setting the stage. The history, chemistry and geobiology behind RNA. in RNA World, Gesteland, R. F., Cech, T. R., Atkins, J. F. editors 1-21
Neveu, M., Kim, H.-J., Benner, S.A. (2013) The "Strong" RNA World hypothesis. Fifty years old. Astrobiology 13, 391-403.

As suggested by the reviewer, we have replaced reference 3 with the references noted above, at the discretion of the editors.

3.1. The central chemical problem for the RNA first model for the origin of life is the discordance between the redox neutral atmosphere that dominated the Hadean, versus the need for reduced nitrogen (at the oxidation state of ammonia) in the nucleobases. The first does not have sufficient ammonia. This is the fundamental flaw with the Sutherland model in reference 1. The compounds that Sutherland invokes in the cyanosulfidic hypothesis are simply not available in any standard model for the Earth. Further, Sutherland's chemistry solves no problem it needs solution. The key step is way of getting formaldehyde, but formaldehyde is abundant in the redox neutral atmosphere without his chemistry. The second key step is joining to formaldehyde units to get C2H4O4, but again, the ppm of this compound that is easily formed in the standard redox neutral atmosphere is adequate to get an autocatalytic cycle to make ribose.

Now, impacts are important parts of actionable models to get RNA, other than Sutherland's, but because they generate reducing atmospheres. The bases in RNA become available only if the atmosphere is reduced by the shattered iron core of an impactor, and that place is a lower limit on the size of the useful impactor it also constraints the latest date when life could have emerged. This is noted on page 5 of the manuscript under review, as well as in reference 21. However, except for people who are very familiar with this field, it is likely to get lost.

We agree with the above and have added a paragraph that directly addresses the role of impacts in the redox problem pointed out by the reviewer. We note that transient reducing atmospheres generated by large impacts (via core disruption and H_2 release; Genda et al., 2017) provide a plausible mechanism for supplying ammonia and other reduced species needed for nucleobase synthesis. We thank the reviewer for emphasizing this point.

Sutherland proposed a different version of Deamer's hypothesis, taking the heavy bombardment effect into account. Instead of hot springs, he considers craters left by meteorite impacts. Rainwater running down their slopes washes out whatever was in the regolith and

whatever the meteorite brought with it. Such a reservoir dries up after a while and then fills with water again.

Our post-4.4 Ga thermal window therefore also corresponds to a period when such episodic reducing conditions could have been productively exploited in stable near-surface niches without subsequent sterilization. This point was under-emphasized in the original submission and is now highlighted.

3.2. Hydrothermal vents are not necessary for any of the actionable models for RNA synthesis. They may be necessary to support a primordial biome, as mentioned on page 6. In fact, all that is necessary for a standard redox neutral atmosphere is a constrained subaerial, intermittently hydrated aquifer. Thus, the prebiotic chemistry line mixes up these two classes of models.

We agree with this point. However, one question that has been repeatedly raised in the literature cannot be overlooked: Did hydrothermal vents contribute to the development of the RNA world? Many workers claim that submarine hydrothermal systems, through their interaction with seawater, generate steep redox gradients (e.g., Ooka et al., 2019) that provide favorable conditions for both the abiotic synthesis of organic compounds and the energetic support of early living systems. It has therefore been proposed that such environments could have facilitated prebiotic organic synthesis. Serpentinization may have supplied reduced nitrogen species which, in the presence of transition metals such as iron and nickel, could have promoted the formation of precursors of nucleic acids, amino acids, and proteins. Authors of this model further suggested that naturally occurring semipermeable compartments within the mineral matrix of hydrothermal chimneys could have functioned as chemoautotrophic protocells subject to natural selection (Koonin & Martin, 2005).

Whether this scenario accurately reflects the origin of life may never be established with certainty. It is worth mentioning that the present manuscript does not advocate or rely on this hypothesis. We have added text clarifying that while impact-induced hydrothermal systems provide energy-rich, disequilibrium environments favorable for early metabolism and mineral catalysis, many prebiotic synthesis pathways (including cyanosulfidic chemistry) can operate in subaerial, intermittently wetted aquifers under redox-neutral conditions. Our thermal-stability analysis maps the necessary physical prerequisite -- persistent mild temperatures -- common to both classes of model.

3.3. Notwithstanding, the major reason why this paper is important, is because it constrains the front end of the process, providing an important analysis that indicates where the window opens, after the probability of a sterilizing impactor has ceased. The discussion of Zircon is important and new.

We are pleased that the reviewer highlights these strengths. No major textual change required, but we have expanded the discussion of the zircon age-resetting in response to comments by other reviewers.

3.4. The molecular clock analysis is fine, although, in Hirakawa (2025) the sequential events relating to origins occurring 100 million years before the clock suggests the divergence of the three kingdoms which occurs 100 million years before the first isotopic. The light carbon reported in Zircon is stated to be "too good to be true". Of course, it may be true.

We have added a note that the results of Hirakawa et al. (2025) suggest prebiotic formation of RNA at ~4.3 Ga, which is fully consistent with our ~4.33 Ga RNA-World sweet spot. Regarding the 4.1 Ga light carbon in zircons (Bell et al., 2015), we retain the citation as "potential" evidence while acknowledging, as the reviewer notes, that it remains debated.

Response to Reviewer #3

We thank Reviewer #3 for the positive evaluation and for highlighting the exciting implication that an RNA World could have emerged significantly earlier than previously assumed, thereby allowing more time for subsequent biological evolution.

Regarding the authors' concept of the RNA world, it is noteworthy that the manuscript continues to treat the molecules required for the origin of life as belonging to two separate domains: RNA and peptides. In 2022, it was proposed that a purely RNA-based world may never have existed, but rather that an RNA-peptide world may have been present from the outset, composed of RNA building blocks that simultaneously carried amino acid functionalities or amino acids themselves. The basis of such a system would be RNA-peptide chimeras. While this distinction is not directly relevant for the publication of the model, it would nevertheless be appropriate to briefly address it in the manuscript.

We thank the reviewer for this valuable suggestion, and we regret not mentioning this explicitly in the original version. Indeed, coevolution of RNA-peptide chimeras introduces new possibilities for the evolution of the origins of life and enables it to adapt to its environment and diversify at an early stage. We appreciate the reference to recent suggestions that a purely RNA World may never have existed and that RNA-peptide chimeric systems were present from the outset.

The primary focus of our study is not to evaluate or distinguish between different hypotheses concerning the origin of life; however, we agree that acknowledging this perspective improves the manuscript. As suggested by the reviewer, we have added a brief discussion on the RNA-peptide world, citing Müller et al. (2022) and clarifying that our framework is compatible with either scenario.

Response to Reviewer #4

We thank Reviewer #4 for raising important points about model heritage, potential improvements, and the role of the zircon analysis. We respond to each concern below.

The manuscript discusses the consequences of terrestrial early bombardments on the origin of life. The writing is clear and well-articulated. However, the study's main conclusions rest on modeling that is quantitatively very similar to work previously published by the same authors (Abramov et al., 2009, 2013). Although the authors have implemented some changes—including the use of different bombardment histories—the overall approach appears to neglect major areas for improvement.

While the core 3-D thermal bombardment code builds on our earlier framework, it is important to note that Abramov and Mojzsis (2009) and Abramov et al. (2013) focused exclusively on the putative period of the "Late Heavy Bombardment" (LHB) at ~3.9 Ga and

did not address late accretion or the origin of life. Abramov and Mojzsis (2009) examined the survival of existing life during the LHB and did not address the origin of life; Abramov et al. (2013) did not provide a biological or habitability analysis.

In contrast, the present study targets the entire late accretion interval and derives the first quantitative timeline for the emergence of the RNA World. In addition, the model of zircon age-resetting in the crust is new and was not part of the earlier papers. Several improvements have also been made to the bombardment model itself, including a much longer run duration (1000 Myr vs. 100 Myr), a bombardment flux that changes with time, and variable impactor velocities.

These changes, together with the coupling of thermal histories to molecule-specific stability thresholds for RNA, DNA, and RNA-DNA complexes, explicit quantification of "never-sterilized" volumes and hydrothermal-cluster statistics via DBSCAN, identification of a precise temporal "sweet spot" at 4.33 Ga for RNA-World emergence, and direct integration with the latest molecular-clock LUCA timeline, make the main conclusions of this study novel and distinct from those of our earlier work. We have added a short paragraph to the paper highlighting these significant advances.

1. The manuscript emphasizes the predicted timing of habitability (~4.3 Ga), which is reasonable based on prior work. However, the modeling presented here relies on scaling laws similar to those used in earlier studies, representing little significant advancement. For instance, the manuscript does not model hydrothermal alteration, which can significantly outlast predictions based on simplistic temperature scaling laws (see the recent Chicxulub debates; Pickersgill et al., 2026). This discrepancy also applies to the relationship between crustal volumes above given temperatures. Moreover, impact melt scaling laws can be improved by following the methodology outlined by Maske et al., 2025.

Although we do not explicitly model hydrothermal activity in the present global study due to computational constraints, we have previously modeled it in detail at individual impact craters, including Sudbury (Abramov and Kring, 2004) and Chicxulub (Abramov and Kring, 2007). We wish to point out that the Pickersgill et al. (2026) study suggested by the reviewer cites our work and generally supports our conclusions. We have added a paragraph to the manuscript describing the effects of impact-induced hydrothermal activity, referring to our previous work and to Pickersgill et al. (2026).

Regarding impact-melt scaling, Manske et al. (2025) compare their new relations to those previously derived by our group (Abramov et al., 2012). In their Figure 8 they note good agreement, with our results essentially bracketing theirs. We have revised the manuscript to add a short discussion on melt scaling and reference both Abramov et al. (2012) and Manske et al. (2025).

2. One critical aspect is the overall delivered mass during late accretion. The manuscript considers masses up to 0.57 wt\% Earth's masses. However, SPH impact simulations have shown that impactor metal retention is not 100% efficient (which is the primary constraint used to derive a late accretion mass), and so a larger mass is likely (Marchi et al., 2018; Citron & Stewart 2022; Itcoviz et al., 2024).

We chose 0.57 wt% as a conservative upper-bound scenario consistent with HSE mantle abundances. The references suggested by the reviewer (Marchi et al., 2018; Citron & Stewart,

2022; Itcovitz et al., 2024) assume that late accretion may have involved mostly large (≥ 1,500 km in diameter) -- and therefore differentiated -- projectiles in which highly siderophile elements were sequestered primarily in metallic cores. In contrast, our simulations assume a size-frequency distribution like the present-day asteroid belt, which, when coupled with delivered mass constraints, implies impactor diameters <~700 km, and thus mostly undifferentiated bodies. This is an important distinction not mentioned in the review.

We agree that delivered mass is a critical parameter and have revised the manuscript to acknowledge the reviewer's point, cite Marchi et al. (2018), expand the discussion of delivered mass, and qualitatively discuss the effects of higher delivered mass on our results.

3. The relevance of the Hadean zircon age reset model to the present paper is unclear. Because the final zircon age distribution is not used to constrain the impact flux, it has no bearing on the biocompatible volume.

We agree that the Hadean zircon age reset model results are not central to our conclusions, which is in large part why we present them mostly in the Supplementary Online Material. That said, the zircon age analysis serves two purposes: (1) as an independent benchmark/validation that the thermal histories generated by our bombardment model are reasonable (for example, if they produced ages significantly younger than those observed, it would flag a problem); and (2) to demonstrate that even under the highest mass flux tested, impact-induced resetting alone produces age distributions older than observed. This mismatch implies that non-impact processes (magmatic zircon crystallization, plate tectonics) were active, which is directly relevant to the persistence of biocompatible niches. We have revised the manuscript to clarify this, but wish to emphasize the importance of testing models such as ours in quantifiable ways if possible.

In summary, we believe these revisions have substantially strengthened the manuscript and have effectively addressed all reviewer concerns. We look forward to next steps.

Sincerely,

Oleg Abramov, Anna Medvegy, Barbara Kremer & Stephen J. Mojzsis
Corresponding authors: abramov@psi.edu; stephen.mojzsis@uni-bayreuth.de
